# Extinction Risk and Diversification Are Linked in a Plant Biodiversity Hotspot

**DOI:** 10.1371/journal.pbio.1000620

**Published:** 2011-05-24

**Authors:** T. Jonathan Davies, Gideon F. Smith, Dirk U. Bellstedt, James S. Boatwright, Benny Bytebier, Richard M. Cowling, Félix Forest, Luke J. Harmon, A. Muthama Muasya, Brian D. Schrire, Yolande Steenkamp, Michelle van der Bank, Vincent Savolainen

**Affiliations:** 1 National Center for Ecological Analysis and Synthesis, University of California, Santa Barbara, California, United States of America; 2 Department of Biology, McGill University, Montreal, Quebec, Canada; 3 South African National Biodiversity Institute, Biosystematics Research and Biodiversity Collections, Pretoria, South Africa; 4 Schweickerdt Herbarium, Department of Plant Science, University of Pretoria, Pretoria, South Africa; 5 Department of Biochemistry, University of Stellenbosch, Stellenbosch, South Africa; 6 Compton Herbarium, South African National Biodiversity Institute, Cape Town, South Africa; 7 Department of Botany and Plant Biotechnology, University of Johannesburg, Johannesburg, South Africa; 8 Bews Herbarium, School of Biological and Conservation Sciences, University of KwaZulu-Natal, Pietermaritzburg, South Africa; 9 Botany Department, Nelson Mandela Metropolitan University, Port Elizabeth, South Africa; 10 Royal Botanic Gardens, Kew, Richmond, Surrey, United Kingdom; 11 Department of Biological Sciences, University of Idaho, Moscow, Idaho, United States of America; 12 Department of Botany and Bolus Herbarium, University of Cape Town, Western Cape, Rondebosch, South Africa; 13 Imperial College London, Silwood Park Campus, Ascot, Berkshire, United Kingdom; The University of North Carolina, United States of America

## Abstract

It is widely recognized that we are entering an extinction event on a scale approaching the mass extinctions seen in the fossil record. Present-day rates of extinction are estimated to be several orders of magnitude greater than background rates and are projected to increase further if current trends continue. In vertebrates, species traits, such as body size, fecundity, and geographic range, are important predictors of vulnerability. Although plants are the basis for life on Earth, our knowledge of plant extinctions and vulnerabilities is lagging. Here, we disentangle the underlying drivers of extinction risk in plants, focusing on the Cape of South Africa, a global biodiversity hotspot. By comparing *Red List* data for the British and South African floras, we demonstrate that the taxonomic distribution of extinction risk differs significantly between regions, inconsistent with a simple, trait-based model of extinction. Using a comprehensive phylogenetic tree for the Cape, we reveal a phylogenetic signal in the distribution of plant extinction risks but show that the most threatened species cluster within short branches at the tips of the phylogeny—opposite to trends in mammals. From analyzing the distribution of threatened species across 11 exemplar clades, we suggest that mode of speciation best explains the unusual phylogenetic structure of extinction risks in plants of the Cape. Our results demonstrate that explanations for elevated extinction risk in plants of the Cape flora differ dramatically from those recognized for vertebrates. In the Cape, extinction risk is higher for young and fast-evolving plant lineages and cannot be explained by correlations with simple biological traits. Critically, we find that the most vulnerable plant species are nonetheless marching towards extinction at a more rapid pace but, surprisingly, independently from anthropogenic effects. Our results have important implications for conservation priorities and cast doubts on the utility of current *Red List* criteria for plants in regions such as the Cape, where speciation has been rapid, if our aim is to maximize the preservation of the tree-of-life.

## Abstract 2

Plant extinction risks in the Cape, South Africa differ from those for vertebrates worldwide, with young and fast-evolving plant lineages marching towards extinction at the fastest rate, but independently of human effects.

## Author Summary

The rapid loss of biodiversity is the most significant ecological challenge we face today. Over the past few years, the International Union for Conservation of Nature has published *Red Lists* documenting the inexorable slide towards extinction of species; recent losses include the Hawaiian crow, golden toad, Baiji dolphin, and the West African black rhino. In groups we know well, such as mammals, the risk of extinction has been related to biology, with the most vulnerable species tending to be large, slow breeding, and narrowly distributed. Although plants are the basis for life on Earth, our knowledge of the drivers of plant extinctions is poor. Here, we disentangle the causes of plant extinctions. We show that the processes linked with extinction risks in plants of the Cape, South Africa differ from those for vertebrates more generally. The most vulnerable species are found within young and fast-evolving plant lineages, opposite to patterns in vertebrates. Our results illustrate the intricate link between the processes of speciation and extinction. We also show that the most threatened species are marching towards extinction at faster rates, but surprisingly, the risk appears independent of human effects.

## Introduction

The rapid and accelerating loss of biodiversity is the most significant ecological challenge we face today. Current rates of extinction are already estimated to be several orders of magnitude greater than background rates [1] and are projected to increase another order of magnitude within the next few hundred years [2]. The terrestrial environment is now dominated by people—approximately one-third of land area has been transformed for human use [3] and one-fourth of global productivity diverted to human consumption [4]. The main direct human-induced drivers that impact biodiversity now are habitat loss and fragmentation, whereas climate change is likely to become a dominant future driver [5]. With each extinction event, we lose an element of biodiversity along with the associated ecosystem services it provides and the unique evolutionary history it represents.

For over four decades the *Red List of Threatened Species* from the International Union for Conservation of Nature (IUCN; http://www.iucnredlist.org/) has provided a record of the incremental slide towards extinction of much of current biodiversity [6]–[8]. Based on detailed, peer-reviewed assessments [9], species are placed into one of the following seven categories, in order of increasing extinction risk: least concern (LC), near-threatened (NT), vulnerable (VU), endangered (EN), critically endangered (CR), extinct in the wild (EW), and finally, extinct (EX). There are currently 47,978 species on the IUCN *Red List*, of which 17,315 are classified as threatened with extinction; the vast majority (75%) of these records are from animals. In vertebrates, including mammals, birds, and amphibians, the proportion of species falling within the different threat categories differs significantly between higher taxa [10]–[13], indicating taxonomic selectivity in species vulnerabilities. Species traits linked with body size, generation times, and geographic range size are commonly associated with threat status [10],[13]–[16], with the most vulnerable species tending to be nested within species-poor clades [12],[13]. Identifying the key traits linked to high extinction risk is critical for predicting future declines and provides an opportunity for implementing preemptive conservation measures [17], although the particular attributes that influence vulnerability can differ among clades and geographic regions [18],[19].

**Figure 1:**
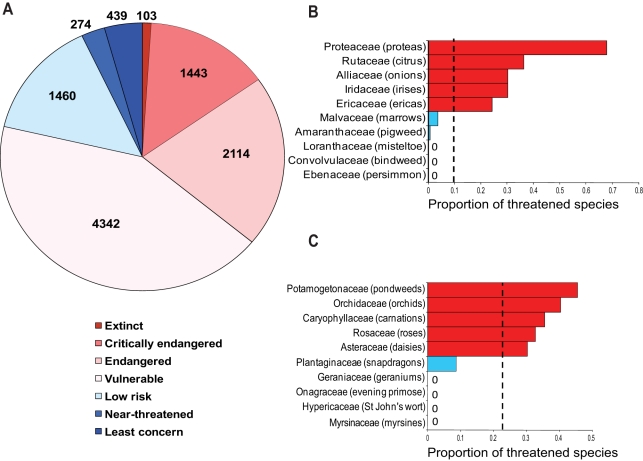
Taxonomic distribution of threat. Global number of angiosperm species listed within each category of the IUCN Red List (A). Comparison of the taxonomic distribution of extinction risk between the South African (B) and the British (C) floras (Tables S3–S10). Families with higher than expected proportions of threatened species are shown in red, and families with significantly lower proportions of threatened species are shown in blue (five most extreme families shown). The dashed line represents the mean proportion of threatened species across all families.

Our knowledge of extinction in plants is much poorer than for vertebrates, with <5% of known plant species assessed (10,916 species) by the IUCN using current criteria. Nonetheless, current listings are still informative, especially if we focus on regions where taxonomic sampling has been more complete. Within flowering plants, over 70% of currently listed species are classified as at risk of extinction (category VU or higher; Figure 1), a much higher percentage than that reported for vertebrate groups (22% listed species). In large part, this likely reflects the relative incompleteness of the dataset for plants, and recent efforts suggest the proportion of threatened plants might be similar to that for mammals [20], although these estimates assume an even distribution of threatened species within higher taxa.

Parallel to trends for vertebrates, plants also demonstrate an uneven distribution of threat across taxonomic ranks in both local floras [21]–[23] and globally, as shown here (*p*<0.001 and *p* = 0.002 from randomizations for families and orders, respectively; see Materials and Methods, Tables S1 and S2) and suggested elsewhere [24]. However, plant studies based on local floras show that life history traits (e.g., pollination syndrome, sexual system, habit, height, and dispersal mode) seem to only correlate poorly with species rarity (relative frequency of occurrence) or threat (index of species' decline or vulnerability to future decline) [25]–[27], and putative key traits differ between studies [23],[26],[28],[29]. Significant taxonomic structure in the distribution of threat in the absence of any strong correlation with heritable biological traits requires an explanation—although it remains possible that important traits have yet to be identified. Further, in contrast to findings for vertebrates, threatened plant species, as indexed by rarity, tend to be over-represented within species-rich taxa [21],. This trend suggests a potential link between the processes of speciation and extinction [30]. We have not attempted to differentiate between rarity and threat status here, because rarity does not have a standard definition or geographical scale and might refer to both local and global scales [21],[30] or relate more directly to extinction risk [21],[22]. Whilst rarity may not always match to estimates of global threat status as defined by the IUCN, it is a central criterion in *Red List* (e.g., Criteria A, C, and D), and regional rarity would be expected to provide a good indicator of regional *Red List* status in the absence of more comprehensive IUCN assessments. Further, a recent study [31] demonstrated that regional *Red Lists* typically correlate strongly with global listings, and for endemic species, regional status will translate directly to global status.

In this study, we explore the distribution of extinction risks for two of the best-studied floras, the Cape of South Africa and the United Kingdom, for which practically complete regional *Red List* data have recently been published (Materials and Methods). These two regions represent very different floristic histories, the former assembled via post-glacial recolonization and range expansion, the latter a global biodiversity hotspot [32] with extraordinary high endemism [33] suggesting a rich history of in situ diversification. If the processes of speciation and extinction are coupled, the Cape flora likely provides the best opportunity for detecting the imprint of any such links. Moreover, if there are similarities in the distribution of extinction risks between the UK and Cape floras, it would be strong evidence for common trends across angiosperms. Here, we contrast the taxonomic distribution of extinction risks between the Cape and the UK floras. We then use detailed phylogenetic data for the Cape to identify the factors likely driving extinctions. Phylogenetic approaches not only allow us to correct for the non-independence of characters given the evolutionary relationships between species but also provide information on species' evolutionary histories. We reveal an unusual phylogenetic signal in extinction risks for plants and demonstrate how species' present-day vulnerabilities can be explained by their recent evolutionary past.

## Results/Discussion

Taxonomic structure in the distribution of threat has provided the stimulus to search for heritable traits that predispose some species towards extinction [12],[34]. If biological traits were the main determinant of extinction risk, and key traits were evolutionarily conserved so that species within clades tended to share similar vulnerabilities, we would predict the taxonomic distribution of threatened species to be broadly similar among regions. We contrasted the distribution of threat between the UK and South Africa. We revealed that taxonomic patterns differ dramatically between these two regions (Figure 1; Tables S3–S10). For example, some taxa show congruent patterns between the two floras (e.g., Brassicales – cabbages and allies), whereas others differ strikingly (e.g., Asterales – daisies and allies). This mismatch indicates that, as shown for other groups [18],[19], geography as well as biology are important in determining vulnerabilities in plants. To disentangle the factors underlying extinction risks we need, therefore, to access not only comprehensive *Red List* data but also detailed information on geography and phylogeny. Uniquely, the Cape of South Africa, a renowned biodiversity hotspot for plant life, provides an ideal case study.

The Cape flora has been the focus of recent *Red List* assessments [35], and a phylogenetic tree depicting the evolutionary relationships among 735 plant genera based upon molecular data is available [36], along with fine-scaled regional distribution records [37]. Here, although we detected an uneven distribution of threatened species across higher taxa, we found no evidence for more closely related lineages to contain similar proportions of threatened species (*p*>0.05 from randomizations using Blomberg et al.'s K-statistic [38]), consistent with the weak correlations between life history traits and species vulnerabilities in plants.

Previous studies have suggested a positive link between rarity and species richness in plants [21],[30]. Using generalized linear modeling (GLM) of threat in genera endemic to the Cape, we show that lineages with a higher proportion of threatened species are not only species rich but also young and rapidly diversifying (*z* = 5.86, *p*<0.001; *z* = −6.99, *p*<0.001; *z* = 5.54, *p*<0.001, from GLMs for richness, age, and diversification, respectively; Table 1). Moreover, in multiple regression including both age and richness as predictor variables, the relationship between threat and species richness is weaker, with taxon age the dominant predictor in the model (partial deviance explained = 0.11 versus 0.17 for richness and age, respectively; Materials and Methods). Therefore, by incorporating information from phylogeny, we demonstrate that the link between richness and threat is in part a likely product of both factors co-varying with clade age, which correlates tightly with diversification rate—younger clades (genera) have diversified at faster rates (Spearman's *rho* = −0.95). Analogous results were obtained when weighting GLMs either by number of listed species within each genus or the ratio of listed to non-listed species within each genus (Materials and Methods; Tables S11 and S12), and when controlling for phylogenetic covariance, but with diversification rate the better predictor (Table S13). Threatened species cluster in young, rapidly diversifying lineages. To evaluate further the link between clade diversification and extinction risks, we then searched for more information on radiating lineages within in the Cape.

**Table 1:** Generalized linear models of extinction risk against species richness, taxon age, and diversification (genera endemic to the Cape of South Africa).

Model	AIC	Explanatory Variable(s)	Coefficient(s)	*z*	*p* Value
1	385.07	species richness	0.512	5.856	<0.001
2	363.72	taxon age	−0.390	−6.99	<0.001
3	390.11	diversification rate	0.0001	5.538	<0.001
4	356.04	species richness	0.294	3.0674	0.002
		taxon age	−0.316	−5.29	<0.001

We identified 11 “Cape clades,” clades thought to have initially diversified in the Cape and with the majority of species indigenous to the Cape Floristic Region [39], for which near-complete species-level phylogenies have been produced previously or for this study (Table 2). For each clade, we used a continuous linear scale between 0 (LC) and 5 (EW) to quantify extinction risks [15]. We partitioned the variance in risk among and between clades to derive an index of the risk disparity through time (DTT) [40]. The disparity value at a given point in time is then the ratio of the average disparity of subclades for which ancestral lineages were present at that time relative to the disparity of all species within the clade [40]. We compared DTT to two alternative null models: first, assuming a Brownian motion model, in which species differences accumulate over time in a manner analogous to a random walk, and second, a punctuated model in which extinction risk was apportioned asymmetrically between daughter species at speciation (Materials and Methods). Although DDT plots are somewhat autocorrelated through time, large within-clade variance at the tips does not necessarily constrain within-clade variance to be low towards the root, or vice versa. Indeed, within the limits defined by the clade origin and the present, it is possible for clades to fall completely above expected values from our null models (e.g., compare DDT for *Disa* to the Brownian expectations).

**Table 2:** Species-level phylogenies for Cape clades.

Clade	Age (my)	Species Sampled (Total in Clade/Cape Species)	Proportion Threatened (Regionally)	Blomberg's K (Brownian Expectations K = 1)
Cypereae [63]	16.81	90 (160/83)	0.13	0.31
*Disa* [64]	12.86	126 (170/92)	0.19	0.14
*Indigofera* [65]	24.02	274 (750/78)	0.05	0.08
*Lachnaea* [66]	15.58	38 (29/29)	0.39	0.53
*Moraea* [67]	6.71	161 (196/173)	0.26	0.40*
*Muraltia* [68]	17.93	76 (115/100)	0.32	0.25
*Pentaschistis* [69]	7.32	80 (90/63)	0.15	0.29
*Podalyrieae* [70]	17.96	107 (128/117)	0.45	0.32
*Protea* [71]	53.94	90 (100/69)	0.48	0.19
Restionaceae^a^^,^ b [72]	62.45	295 (420/180)	0.16	0.02*
*Zygophyllum* [73]	2.18	61 (132/29)	0.09	0.10

a Branch lengths transformed to make tree ultrametric using penalized likelihood as implemented in the APE R-library [74].

b Restionaceae demonstrates significant phylogenetic signal from randomizations, but the very low K-value (0.02) suggests the covariation with phylogeny is weak.

*p<0.01.

**Figure 2:**
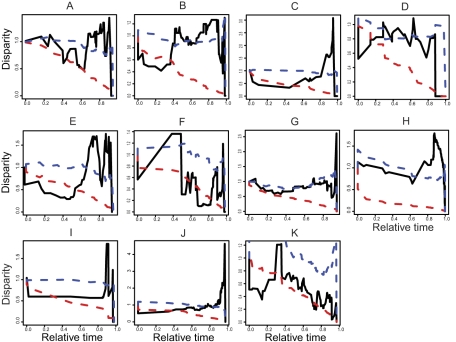
Disparity through time in extinction risk. (A) Cypereae, (B) *Disa*, (C) *Indiogofera*, (D) *Lachnaea*, (E) *Muraltia*, (F) *Pentaschistis*, (G) *Podalyrieae*, (H) Restionaceae, (I) *Zygophyllum*, (J) *Protea*, and (K) *Moraea*. Solid line, observed values; dashed red line, Brownian expectations; dashed blue line, punctuated model (range asymmetry factor  = 2, s.d. for evolutionary drift = 2, evolutionary trend  = 0.3; Materials and Methods). Clade age is scaled between 0 and 1, with 0 zero representing clade origins and 1 representing the present day. High relative disparity towards the present (e.g., Cypereae, *Indigofera*, *Muraltia*, *Podalyrieae*, *Protea*, and Restionaceae) indicates that most variation in extinction risk is between species within subclades, i.e. closely related species frequently differ strongly in extinction risk. Low relative disparity towards the root of the tree (e.g., *Disa*, *Indigofera*, *Moraea*, and *Muraltia*) indicates that most variation in extinction risk is between subclades, i.e. species are more similar in extinction risk within subclades than between subclades.

A Brownian motion model of trait change was strongly rejected (Table 2, Figure 2), and biologically significant phylogenetic signal in threat was only detected within *Moraea* (K = 0.39 versus an expectation of K = 1.0 for a Brownian motion model and K = 0 for absence of phylogenetic signal; see Materials and Methods). These results are inconsistent with a simple, heritable, trait-based model of extinction, although it remains possible that the important traits influencing extinction risks evolve in a non-Brownian fashion. However, we observed two common trends. First, most variation in extinction risk was between species at the tips of the phylogenetic trees—disparity within clades above the line derived from Brownian expectations (Figure 2) (e.g., Cypereae, *Indigofera*, *Muraltia*, *Podalyrieae*, *Protea*, and Restionaceae). Second, towards the root of the tree more variation in risk was observed between clades, disparity within clades being below the line derived from Brownian expectations (Figure 2) (e.g., *Disa*, *Indigofera*, *Moraea*, and *Muraltia*). The distribution of extinction risk therefore can be described as fitting a late-burst model of evolution, in which threat is phylogenetically conserved within deep (early diverging) clades but differs between closely related species at the tips of the tree. What process might explain this unusual phylogenetic distribution of extinction risks?

In vertebrates, the least threatened taxa are found in the more diverse clades [12],[13], which might be expected from a simplistic assumption that low extinction elevates net diversification (speciation – extinction) rates [12]. We have shown that in plants, the pattern is reversed—more rapidly diversifying clades have more vulnerable species. We suggest this contrast between plants and animals reflects differences in their predominant mode of speciation. Speciation in plants is often associated with the establishment of small, reproductively isolated populations, for example, via occasional long-distance dispersal, flower-pollinator co-evolution, hybridization, and polyploidization [41]. Because small range size is a key IUCN criterion for assessing *Red List* status [9], rapidly diversifying lineages will then tend to have a high proportion of threatened species. Further, under a model of peripatric speciation, the geographic ranges of recently diverged taxa are also predicted to display large asymmetry in range size [42], explaining large differences in threat status among recently diverged species, and consistent with the late-burst evolutionary model suggested from the DTT plots.

Our simulations assuming punctuated evolution capture well the asymmetry in trait values predicted from a peripatric model of speciation and better predict the disparity between species within clades than the Brownian null (Figure 2). To evaluate more directly the mode of speciation, we repeated the DTT plots with range size as the trait of interest. We show a strikingly similar trend for large disparity within clades towards the tips of the phylogenetic trees (Figure S1), fitting well the peripatric speciation model. However, less apparent is any trend for greater between-clade disparity towards the root of the trees (exception *Moraea*), consistent with evidence showing only weak phylogenetic signal in range size [43],[44].

**Figure 3:**
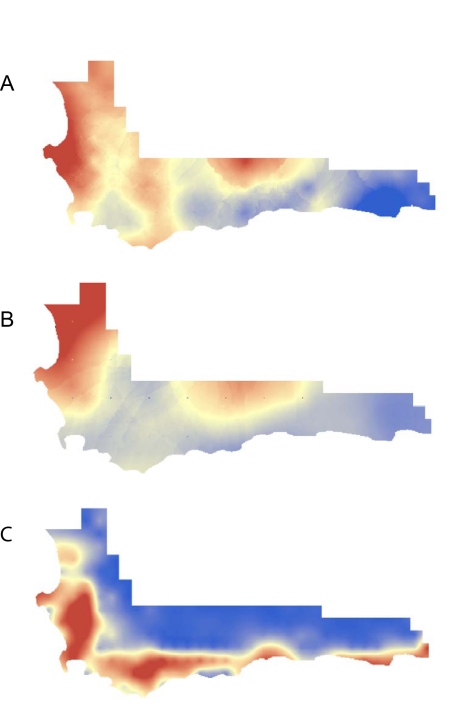
Geography of threat in the Cape of South Africa. Threat (A) correlates with diversification rates (B) rather than habitat transformation (C). Colors reflect interpolated values derived from QDS cell centre points using Ordinary Kriging with a 12-cell neighborhood and scaled from high (red) to low (blue). Point estimates for threat and rates from mean values for endemic genera.

If threat in plants is a product of the speciation process, we might predict extinction risk to be largely independent from anthropogenic drivers, such as habitat loss, which would have important implications for how we manage the conservation of threatened species. We tested this hypothesis using detailed taxonomic distribution data for the Cape [37] and an index of habitat transformation that aggregated the combined impacts of urbanization, cultivation, and alien invasion [45]. We found significant geographic clumping of threat even after correcting for taxonomic richness (Moran's I = 0.07, *z* = 10.11, *p*<0.01; Figure 2). However, “extinction hotspots” did not correlate with habitat transformation (Pearson's *r* = 0.038, *F* = 0.065, *p* = 0.8, adjusting degrees of freedom to correct for spatial autocorrelation; Figure 3), suggesting that the current threat status of Cape plants is independent from anthropogenic drivers, although that is not to say that they might not be important in the future. In contrast, but consistent with our findings above, we find that “extinction hotspots” reflect locations where lineages have recently diversified (Pearson's *r* = 0.504, corrected *F* = 0.534, corrected *p*<0.001; Figure 3).

Finally, we looked at the trend in species' risk status over recent years. Because we found that threatened species often represent recently diversified taxa, we might expect over time young species to expand their geographic distributions as they become established and, as a consequence, decrease in perceived vulnerability. If this was the case, IUCN *Red List* classifications may be misleading, erroneously listing species with small but potentially expanding distributions. However, by comparing consecutive *Red Lists* of the South African flora (Materials and Methods), we found that the most threatened taxa are marching towards extinction at the fastest pace (ratio of species increasing in threat status against those remaining unchanged or decreasing in status: 43∶44 versus 28∶62 for genera within the top and bottom tertiles, respectively, ranked using 1996 *Red Lists*: G-test: G = 12.61, *p*<0.001). The species identified as most vulnerable by the IUCN *Red List* appear firmly committed to extinction. Our results suggest extinction hotspots may therefore represent both cradles and graveyards of diversity—linking the processes of speciation and extinction [46],[47].

Our results explain the paradox of strong phylogenetic structure in extinction risk in the absence of biological predictors at the species level. Speciation via peripheral isolates will result in asymmetry in range size—and therefore threat status—between closely related species that tend to share similar suites of biological traits. However, differences among lineages in the propensity to diversify will result in deeper phylogenetic structure of threat. Our simulations, assuming asymmetry in daughter lineages, fit well the observed disparity in species values within clades. However, we did not replicate the trend for deeper phylogenetic structure because we made the simplifying assumption that diversification rates were independent from trait values (a necessary limitation allowing us to simulate trait evolution along the branches of the empirical set of phylogenies). Finally, because diversification is also influenced by locations, traits that predispose species to diversify in one environment may fail to do so in another [48],[49], explaining regional variation in both threat and species richness.

### Conclusion

As we move towards assembling the complete tree-of-life, there has been increasing emphasis on preserving phylogenetic diversity [50],[51]. Recently, Vamosi and Wilson [24] suggested that globally we risk losing a disproportionate amount of angiosperm evolutionary history as we lose the most vulnerable species to extinctions. Using more detailed phylogenetic information, we show that plant extinctions may result in little loss of evolutionary history, at least in biodiversity hotspots where much of present-day diversity is a product of recent speciation. It is possible that the processes driving extinction in relict lineages are different from those for young diversifying lineages and that there exist two classes of globally threatened plant species. Estimating the true impact of plant extinctions on the loss of evolutionary history across the angiosperm tree-of-life will therefore require detailed knowledge of the interspecific phylogenetic relationships within higher taxa. Our results linking speciation and extinction derive from an analysis of the unique flora found within the Cape region of South Africa and might not extrapolate across less diverse biomes with different evolutionary histories. However, we note that the trend for threatened species to fall within species-poor clades has been observed within the relatively depauperate floras of the UK [22] and North America [21]. We suggest that species turnover (speciation and extinction) might be rapid generally for plants, and high extinction rates may not be unusual over evolutionary timescales. Because even rapid speciation occurs over timescales too long for practical management [52], conservation efforts must focus urgently on reducing rates at which species are being lost [53]–[55]. However, if we wish to maximize the preservation of the tree-of-life [11], we must consider whether plants and animals may be best served by different assessment criteria when deciding upon conservation priorities. For example, for plants, investing in currently less threatened but still vulnerable species in more evolutionary distinct clades might be the most sensible conservation strategy, whereas for vertebrates the IUCN *Red List* may provide a more straightforward index for conservation decision making.

## Materials and Methods

### Taxonomic and Phylogenetic Data

Plant species *Red List* data were extracted from the following sources: (1) Global data: The IUCN Red List of Threatened Species (http://www.iucnredlist.org, accessed August 2009); (2) UK flora: Joint Nature Conservation Committee's Vascular Plant Red Data List for Great Britain 2006 (http://www.jncc.gov.uk); and (3) South African flora: the Interim Global Status for plants published online by the South African National Biodiversity Institute (SANBI: http://www.sanbi.org/; accessed June 2008), and the Beta version of South African Plant Red List including all assessed taxa and their status (SANBI: http://www.sanbi.org/; accessed October 2010). An updated print version is now available from the Pretoria National Botanical Garden [35].

Plant names were synonymized to agree with the Angiosperm Phylogeny Group [56] taxonomy.

We used IUCN *Red List* categories to classify each listed species as either threatened (vulnerable [VU], endangered [EN], critically endangered [CR], extinct in the wild [EW], and extinct [EX]) or non-threatened (least concern [LC] and near-threatened [NT]). Taxonomic structure was evaluated by recording the ratio of threatened to non-threatened species recorded within higher taxa (families, orders, and, for the local floras, APG [56] higher taxonomic class) and calculating the variance across taxa. Significance was assessed by randomizing species membership among taxa and recalculating the ratio of threatened species within each random assemblage, keeping number of species per taxon constant. Taxa with significantly more or less threatened species were identified by comparing the observed proportion of threatened species with expectations from the randomizations. The *p* values were determined from 1,000 random draws.

Last, we synonymized 1996 [57] and 2007 *Interim Global Status* for the South African flora so that extinction risk categories were broadly equivalent, and we scored species included in both listing as +1 if threat increased or −1 if threat decreased. We then derived an index of change in threat status by summing species values within each genus. Because criteria used in the 1996 and 2007 listing were not directly comparable, we evaluated whether species identified as the most vulnerable to extinction in the 1996 listing were more or less threatened in the subsequent assessment by contrasting trends between the top third most threatened taxa with trends within the bottom third (least threatened taxa), ranked using the 1996 listing.

We quantified phylogenetic signal in extinction risk for the Cape flora using Blomberg's K-statistic [38] on the recent comprehensive phylogenetic tree of Cape genera [36]. We use the proportion of threatened species within genera, as described above, as our index of threat. Significance was calculated by randomizing the tips of the tree and recalculating K (1,000 replicates). The K statistic compares the distribution of phylogenetically independent contrasts across nodes within the clade [58], to expectations under a Brownian motion model of trait evolution. Because our metric of extinction risk is bounded between 1 and 0 and therefore violates assumptions of normality, we report only the *p* values from randomizations.

Within the Cape flora, we characterized the phylogenetic distribution of extinction risk across 11 exemplar clades resolved at the species level (Table 2). For each clade we then described variance in extinction risk between and within clades using Harmon and colleagues' index of disparity through time (DTT) [40]. DTT is derived from the standardized mean pair-wise distance between species and therefore does not necessitate the reconstruction of ancestral states. Values of DTT near 0 indicate that most of the variation is partitioned between clades, whereas values near 1 indicate that most variation is among species within subclades. Because at the limits DTT must be 1 at the root and 0 at the tips, we compared observed values to two alternative null models. First, we derived expectations under a null model of Brownian motion. Second, we simulated a model of punctuated evolution in which daughter lineages are assigned trait values asymmetrically at speciation. Following divergence, one daughter lineage inherits a value that differed from the parental lineage by some constant factor, and the other daughter lineage assumes the parental value. Prior to subsequent diversification, both lineages evolve trait distances with a drift factor taken from a normal distribution with mean zero and a given standard deviation plus an evolutionary trend for the smaller lineage to expand in size. This latter simulation might be considered to approximate a model of range size evolution assuming speciation via peripheral isolates, in which one daughter lineage has restricted initial geographic distribution. To evaluate the link between speciation mode and extinction risk directly, we then repeated the analysis of DTT using range size as the trait of interest. Here we estimate range size as the number of quarter degree squares with presence data for each species [37]. If speciation via peripheral isolates is common, we would expect large disparity in range size between species within clades towards the tips of the phylogeny. We did not attempt to link speciation and extinction rates with trait values because to compare DTT plots phylogenetic topology must be identical. Simulations were implemented in the Geiger R-library [59]. R-code for the punctuated model is available from the authors.

### Regression Models

For all Cape genera we compiled data on species richness (n) [60], time to most recent common ancestor (millions of years; my), and net diversification rates (log[n]/my). We then constructed a series of regression models to describe the relationships between threat and taxon age, richness, and diversification rate. Statistical models were constructed in the R statistical package (http://www.r-project.org/). First, we generated a series of single-predictor generalized linear models (GLMs) with the proportion of threatened species in each genus as the response and assuming binomial errors. Species richness and diversification rates were log transformed, and taxon ages square-root transformed prior to model fitting. Second, we constructed a two-predictor model including both species richness and age as explanatory variables. Model fits were assessed using Akaike's information criterion. The marginal contributions of each variable in the two-predictor model were estimated as the additional percent deviance explained by inclusion of that variable to the reduced model. Because missing lineages might result in overestimation of taxon ages and hence underestimation of diversification rates, we focused our analyses on genera endemic to the Cape. Cape endemics are most likely to have their sister clades also included in the phylogenetic tree, and therefore subtending branches will not be broken by the addition of missing taxa; nonetheless, models including all Cape genera also supported the significant relationship between taxon age and threat (not shown).

We evaluated model sensitivity in two ways. First, we repeated the set of regression models weighting the data by (1) the logarithm of the number of *Red List* records for each genus, thereby assuming our estimates of threat are more reliable for genera where multiple species have been assessed, although this will also down-weight the influence of species-poor taxa, and (2) the ratio of listed species to total clade species richness, providing an indication of the impact of missing (unlisted) species. Second, we generated a phylogenetic distance matrix (√my) and used partial Mantel tests to determine the relationship between threat and diversification whilst controlling for phylogenetic relatedness.

### Geographic Analyses

We extracted the list of genera within each quarter degree square in the Cape from the PRECIS database [37]. We then calculated the mean proportion of threatened species and mean diversification rate for genera within cells, as well as a per-cell index of human impact on the environment [45]. Spatial correlation coefficients were calculated using Pearson's correlation coefficient (r) for grid cells and controlling for spatial covariance when estimating the statistical significance by adjusting degrees of freedom [61]. First, we estimated the correlation strength between mean threat and human impact, because habitat transformation is thought to be a key driver of species extinctions. Second, we estimated the correlation strength between threat and diversification, because this was the best of our predictor variables from the regression models (above). Spatial analysis was performed in ArcMap (9.2 Environmental Systems Research Institute Inc.) and SAM (Spatial Analysis in Marcoecology v3.0) [62].

## Supporting Information

Figure S1 Disparity through time in range size.
(0.03 MB PDF)

Table S1 IUCN *Red List* data summarized for angiosperm families.
(0.03 MB PDF)

Table S2 IUCN *Red List* data summarized for angiosperm orders.
(0.02 MB PDF)

Table S3 South African families.
(0.03 MB PDF)

Table S4 South African orders.
(0.02 MB PDF)

Table S5 South African APG taxonomic class 4.
(0.02 MB PDF)

Table S6 South African APG taxonomic class 5.
(0.02 MB PDF)

Table S7 UK families.
(0.03 MB PDF)

Table S8 UK orders.
(0.02 MB PDF)

Table S9 UK APG taxonomic class 4.
(0.02 MB PDF)

Table S10 UK APG taxonomic class 5.
(0.02 MB PDF)

Table S11 Generalized linear models of extinction risk against species richness, taxon age, and diversification (genera endemic to the Cape of South Africa), weighted by number of listed species within each genus (compare with Table 1, main text).
(0.02 MB PDF)

Table S12 Generalized linear models of extinction risk against species richness, taxon age, and diversification (genera endemic to the Cape of South Africa), weighted by the ratio of listed species to total clade species richness within each genus (compare with Table 1, main text).
(0.02 MB PDF)

Table S13 Correlation coefficients between extinction risk, species richness, and taxon age, from Mantel tests controlling for phylogenetic non-independence among genera (compare with GLMs, Tables 1, S11, and S12).
(0.02 MB PDF)

## Abbreviations

CR: critically endangered
DTT: disparity through time
EN: endangered
EW: extinct in the wild
EX: extinct
GLM: generalized linear modeling
IUCN: International Union for Conservation of Nature
LC: least concern
NT: near-threatened
VU: vulnerable
