# Supplementary material for: Extinction Risk and Diversification Are Linked in a Plant Biodiversity Hotspot
Source: PLoS Biol. 2011 May 24;9(5):e1000620. doi: 10.1371/journal.pbio.1000620 (PMC3101198; doi:10.1371/journal.pbio.1000620)
Supplement: Figure S1 — Disparity through time in range size. (0.03 MB PDF) [file pbio.1000620.s001.pdf]

Figure S1. Disparity through time in range size. a) Cypereae, b) *Disa*, c) *Indiogofera*, d) *Lachnaea*, e) *Muraltia*, f) *Pentaschistis*, g) *Podalyrieae*, h) Restionaceae, i) ,*Zygophyllum*, j) *Protea*, k) *Moraea*. Solid line = observed values, dashed red line = Brownian expectations, dashed blue line = punctuated model (range asymmetry factor = 2, s.d. for evolutionary drift = 2, evolutionary trend = 0.3; Materials and Methods). Clade age is scaled between 0 and 1, with 0 zero representing clade origins, and 1 representing the present day. Compare with Figure 2.

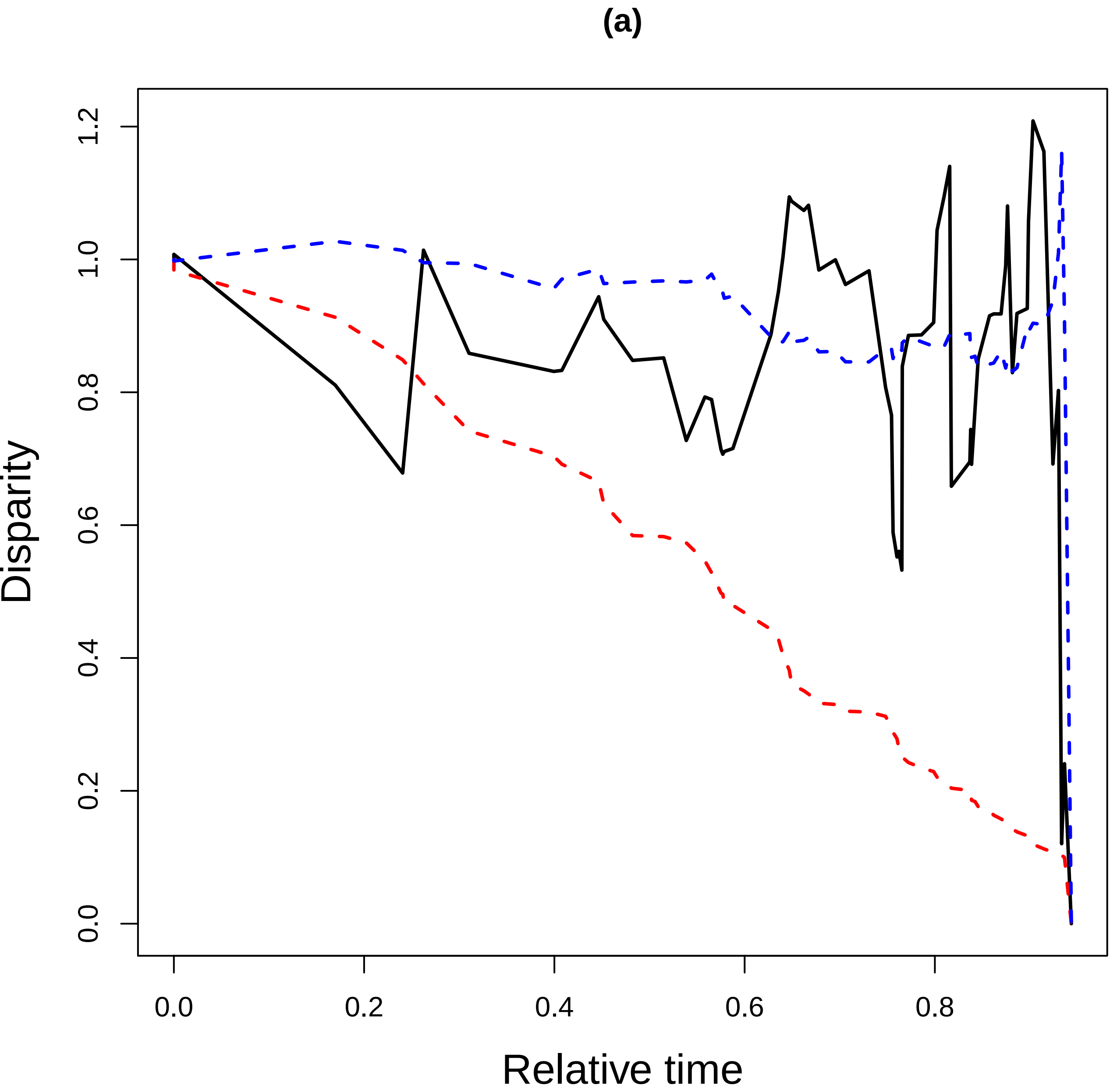

(a)
Disparity
Relative time
0.0
0.2
0.4
0.6
0.8
1.0
1.2
0.0
0.2
0.4
0.6
0.8

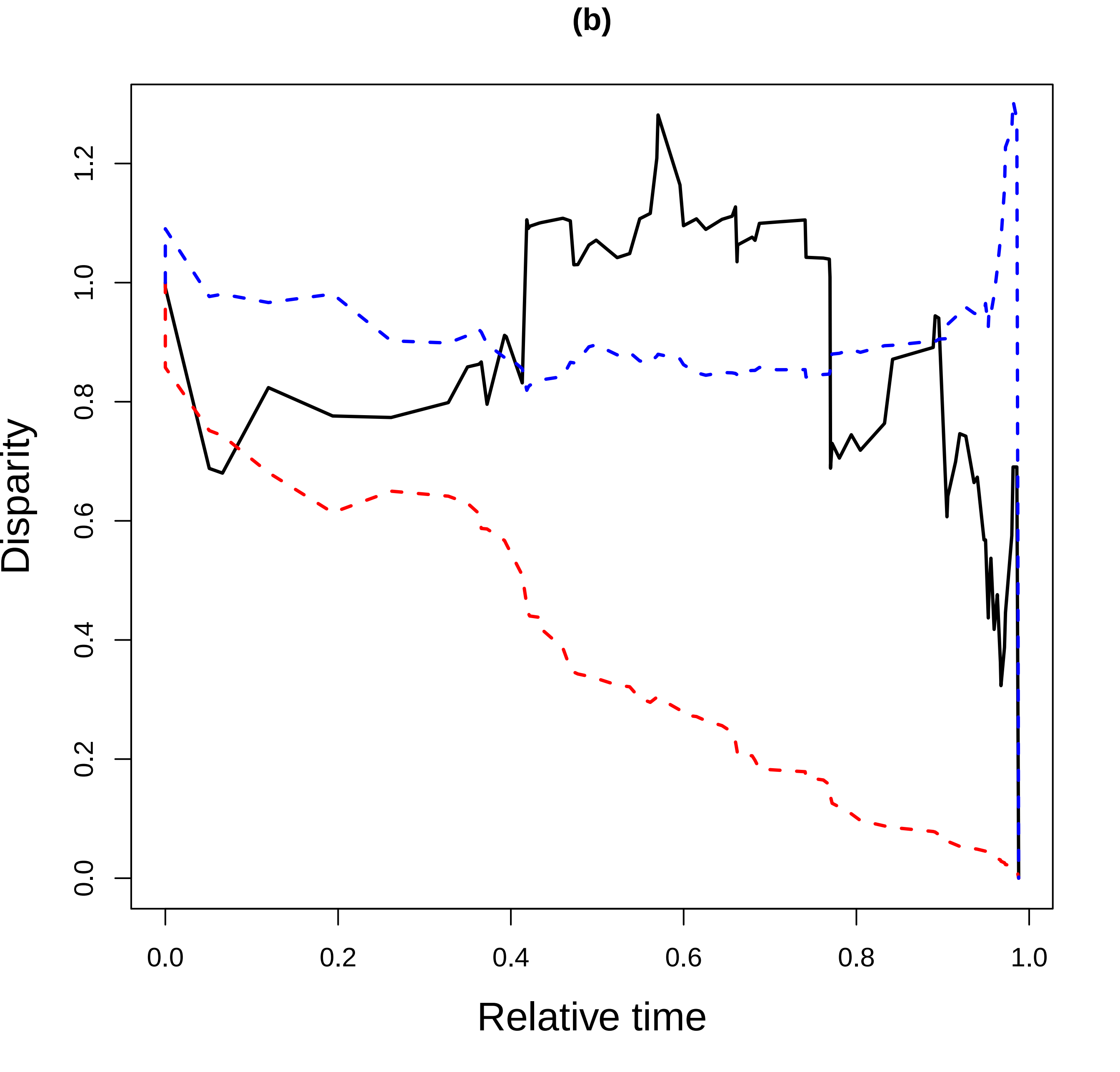

(b)
Disparity
Relative time
0.0
0.2
0.4
0.6
0.8
1.0
1.2

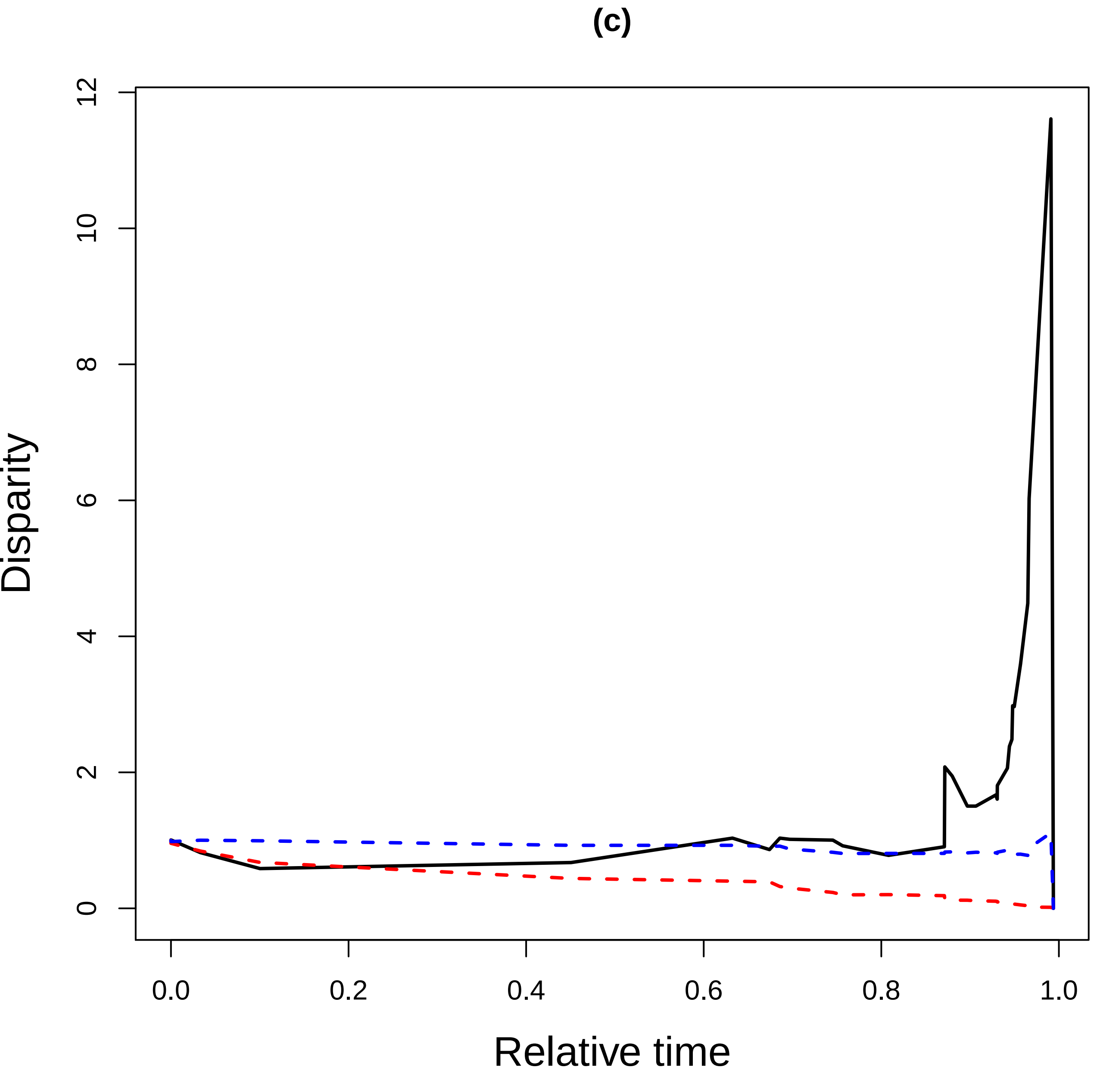
(c)
Disparity
0
2
4
6
8
10
12
0.0
0.2
0.4
0.6
0.8
1.0
Relative time

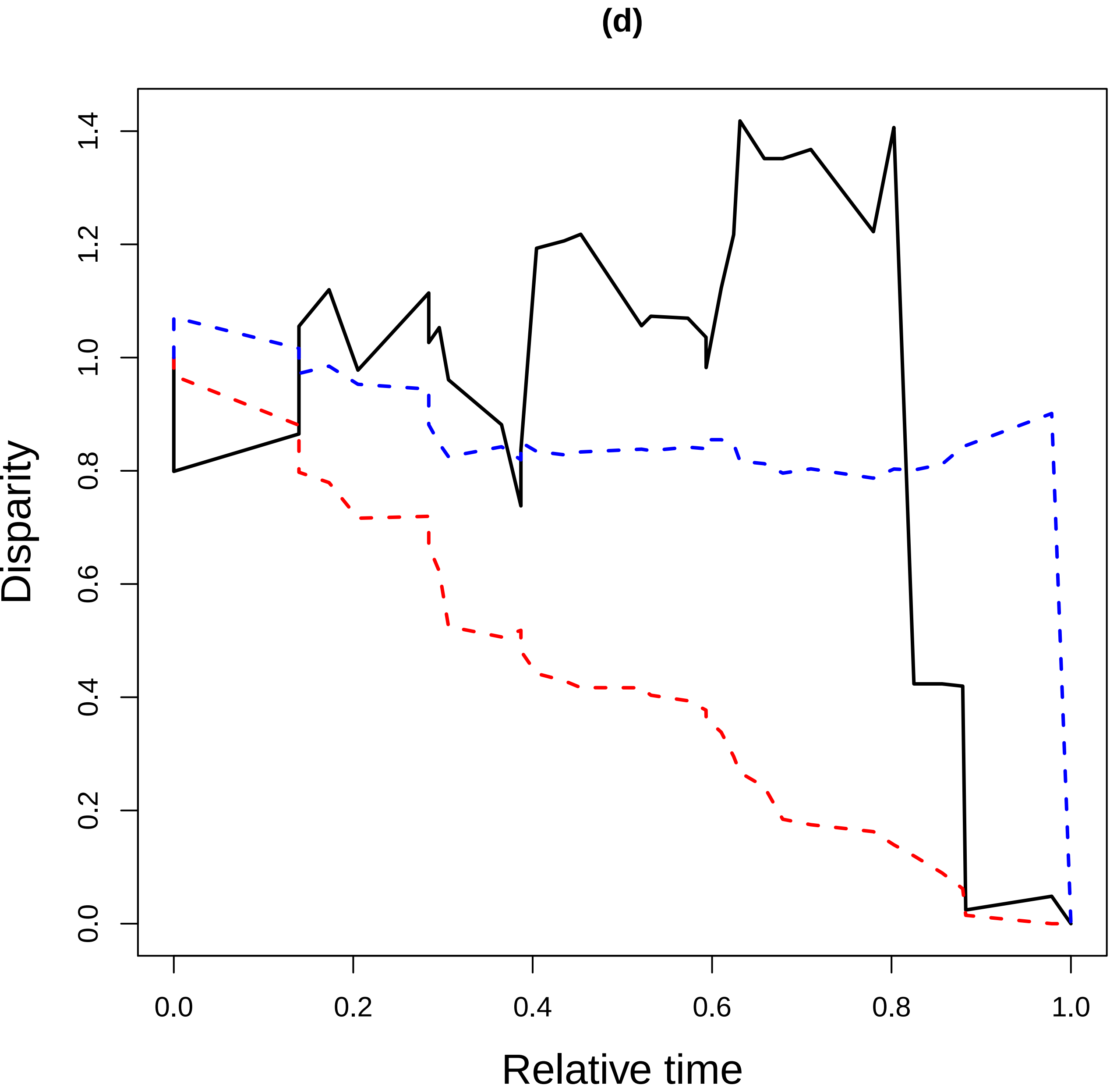

(d)
Disparity
Relative time
0.0
0.2
0.4
0.6
0.8
1.0
1.2
1.4

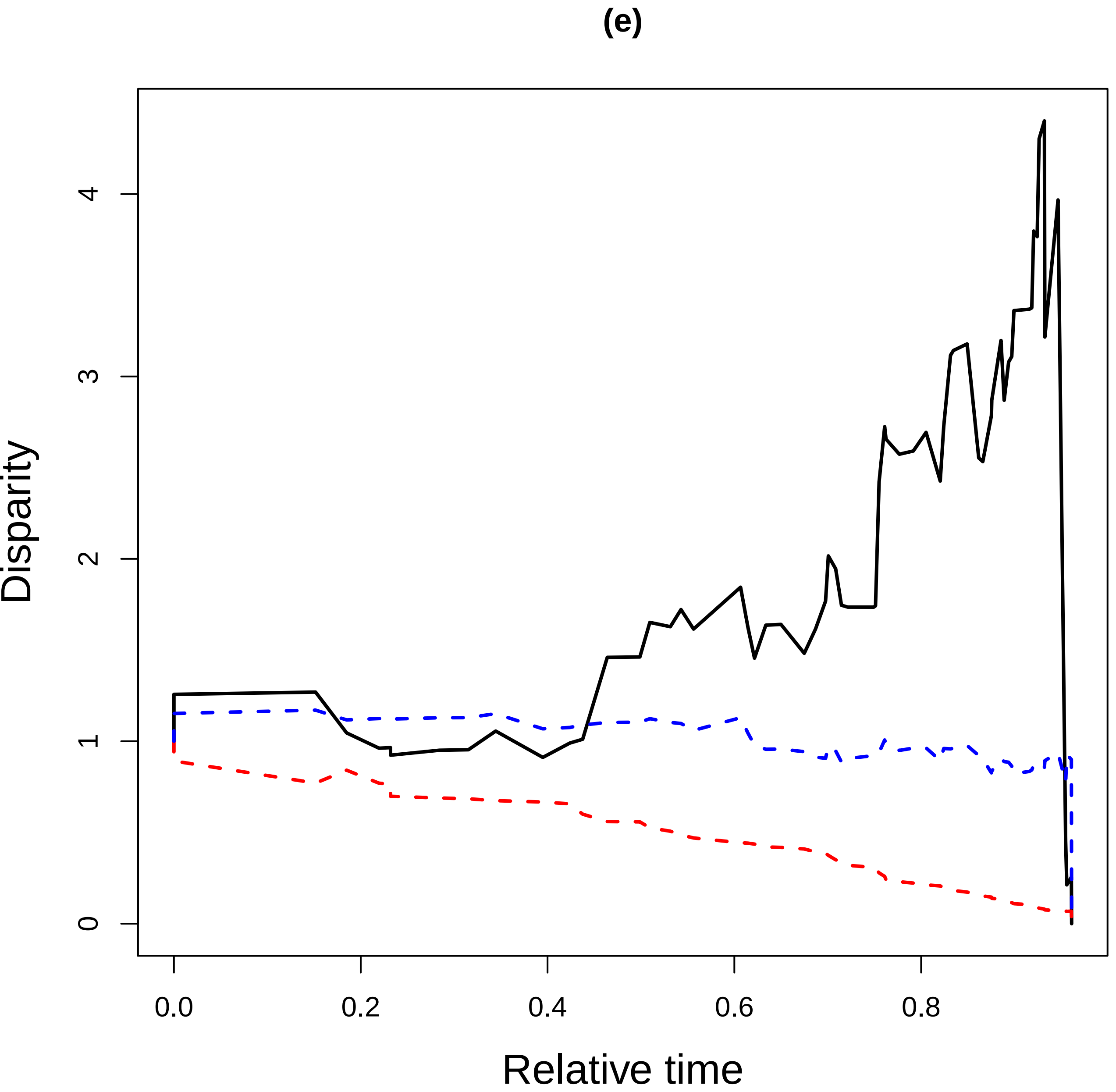

(e)
Disparity
0
1
2
3
4
0.0
0.2
0.4
0.6
0.8
Relative time

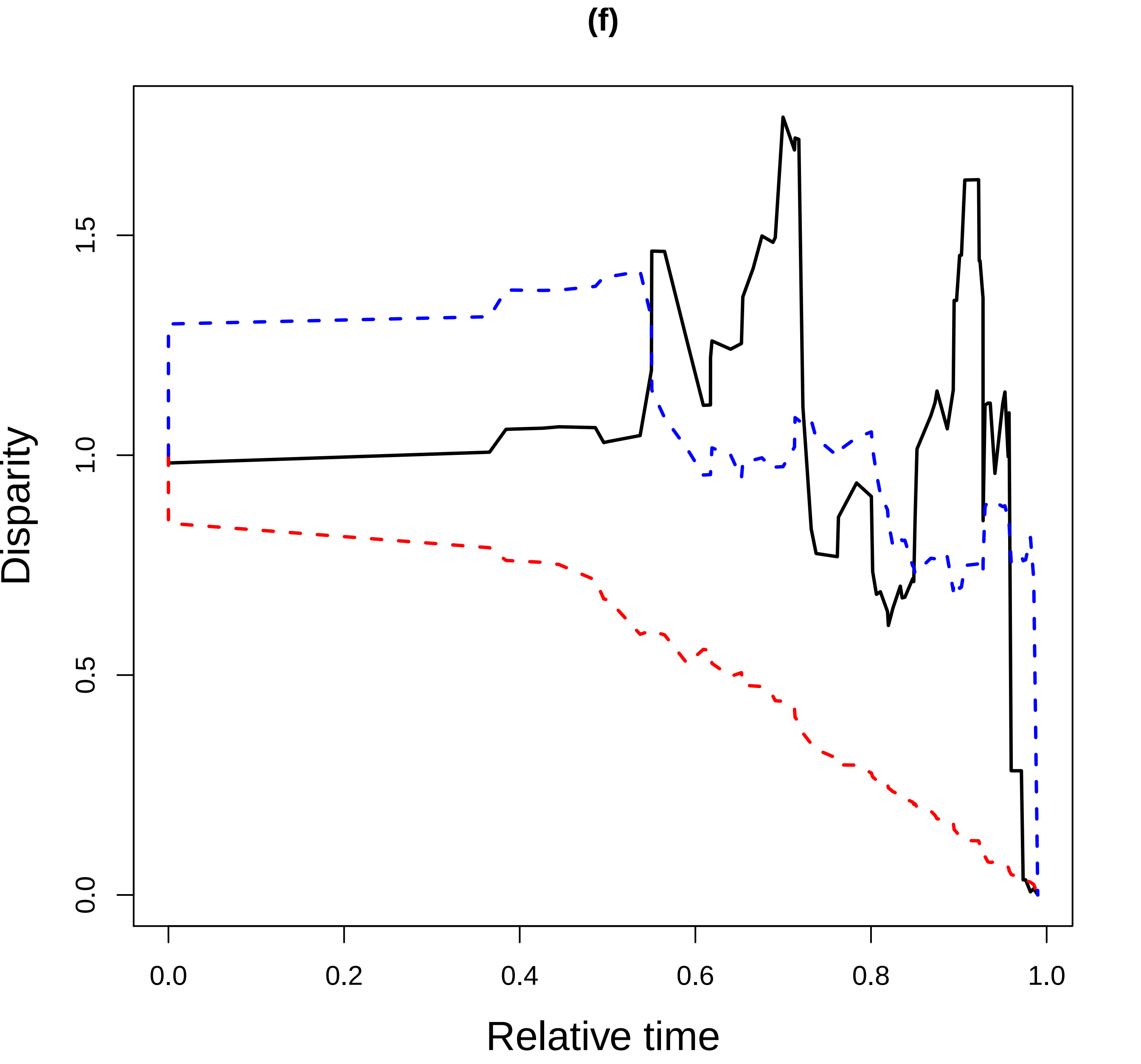

(f)
Disparity
0.0
0.5
1.0
1.5
0.0
0.2
0.4
0.6
0.8
1.0
Relative time

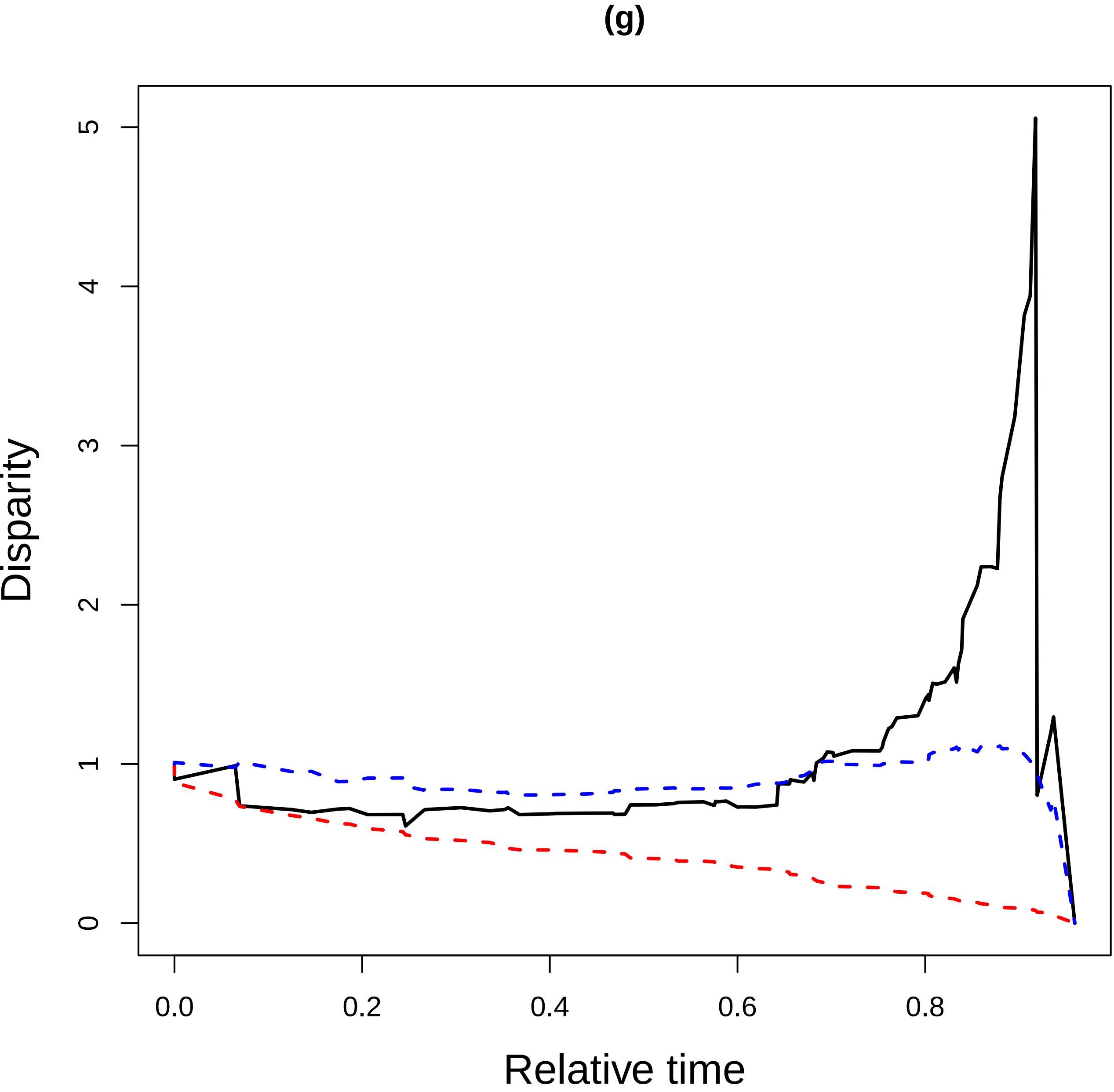

(g)
Disparity
0
1
2
3
4
5
0.0
0.2
0.4
0.6
0.8
Relative time

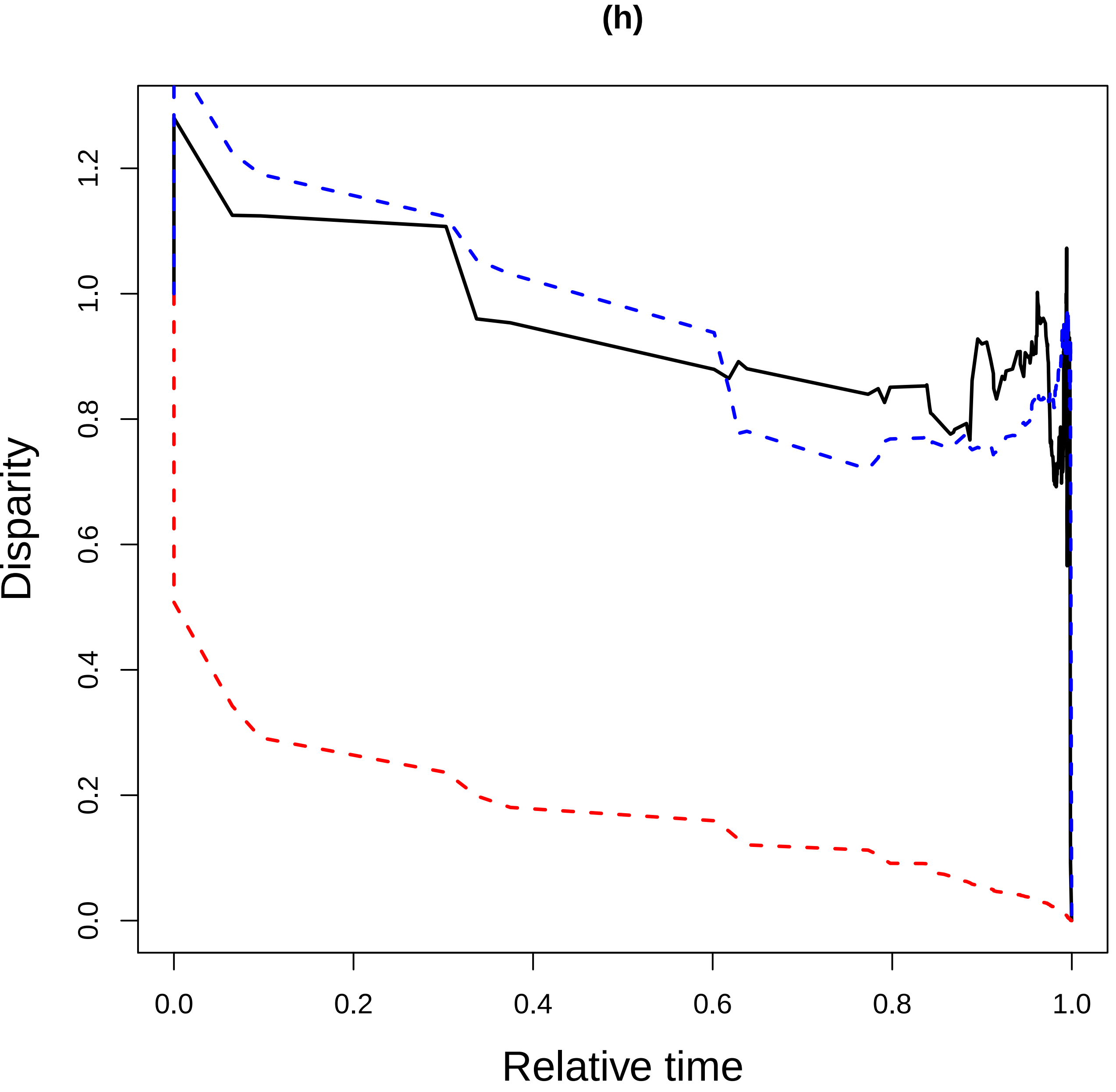

(h)
Disparity
0.0
0.2
0.4
0.6
0.8
1.0
1.2
Relative time
0.0
0.2
0.4
0.6
0.8
1.0

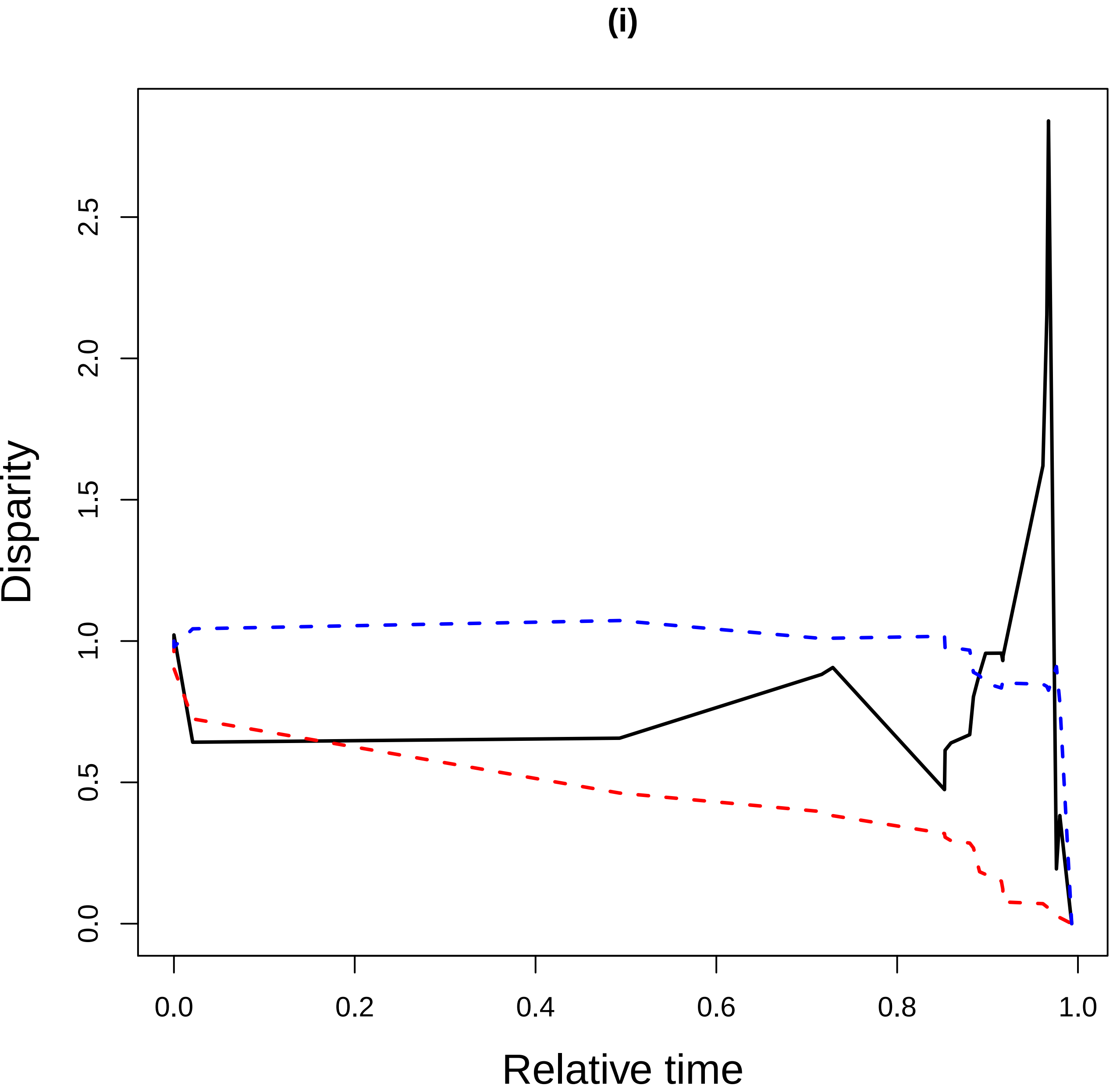

(i)
Disparity
Relative time
0.0
0.5
1.0
1.5
2.0
2.5
0.0
0.2
0.4
0.6
0.8
1.0

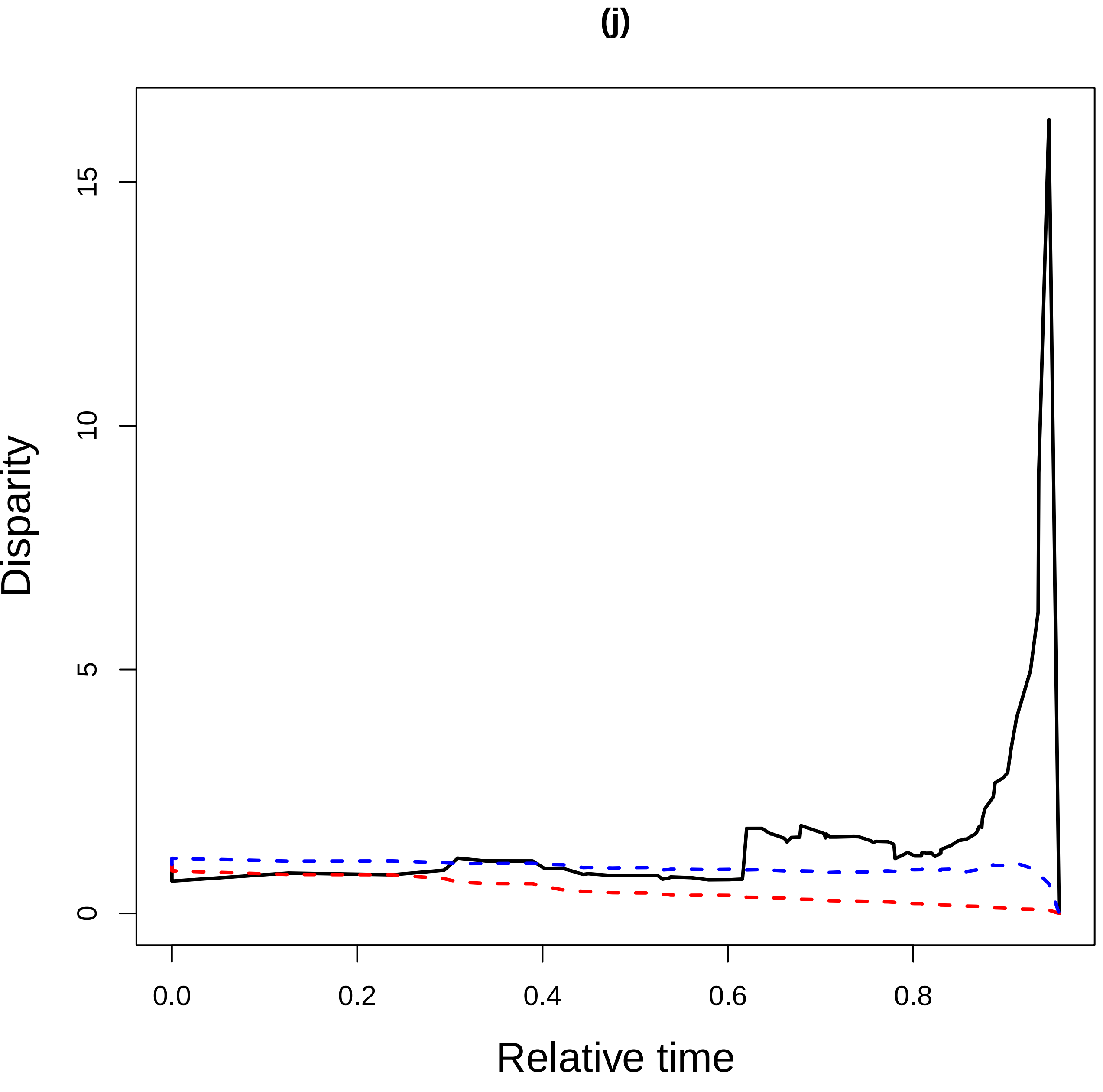
(j)
Disparity
0
5
10
15
0.0
0.2
0.4
0.6
0.8
Relative time

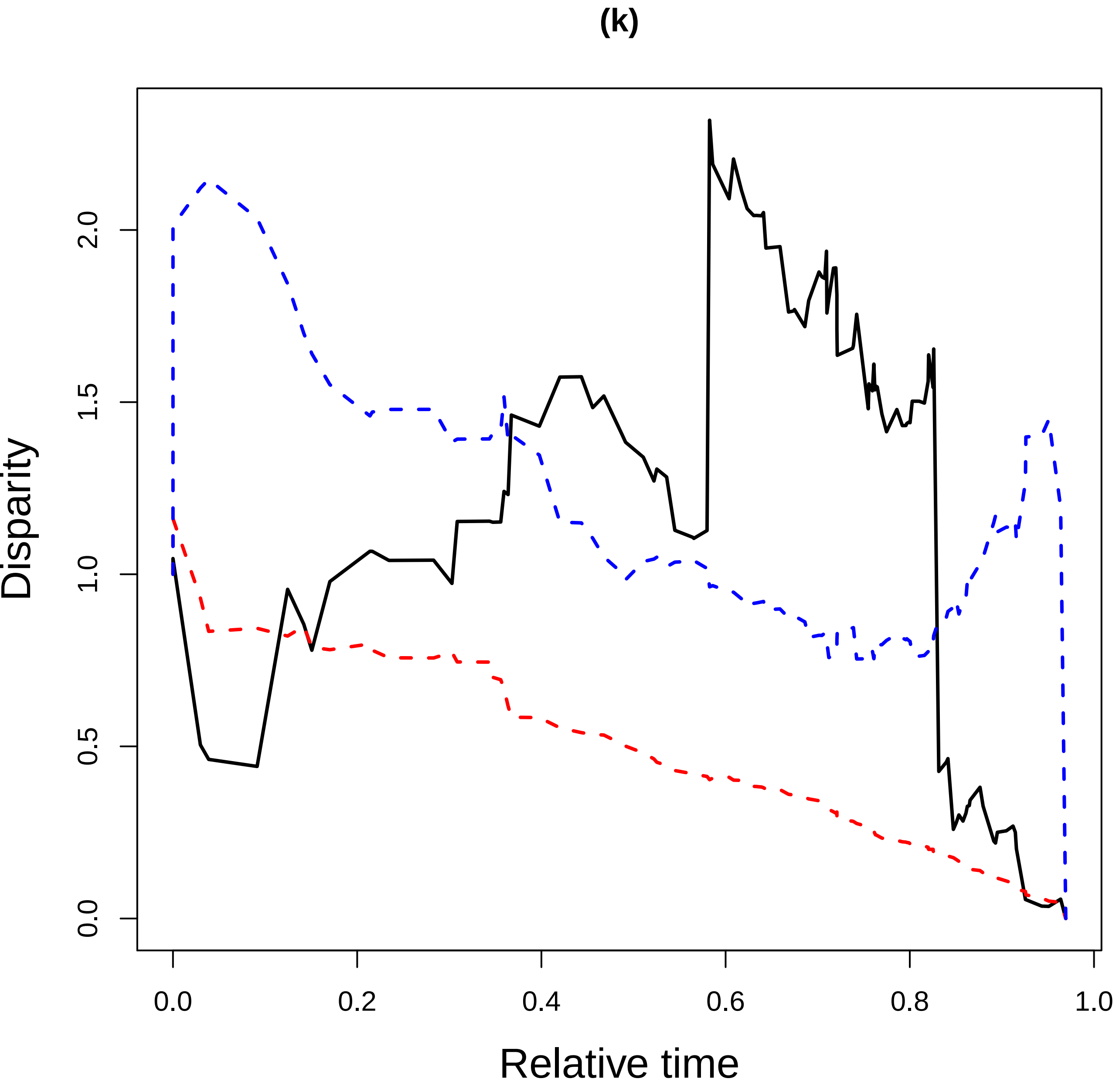

(k)
Disparity
2.0
1.5
1.0
0.5
0.0
0.0
0.2
0.4
0.6
0.8
1.0
Relative time
