# Supplementary material for: Extinction Risk and Diversification Are Linked in a Plant Biodiversity Hotspot
Source: PLoS Biol. 2011 May 24;9(5):e1000620. doi: 10.1371/journal.pbio.1000620 (PMC3101198; doi:10.1371/journal.pbio.1000620)
Supplement: Table S1 — IUCN Red List data summarized for angiosperm families. (0.03 MB PDF) [file pbio.1000620.s002.pdf]

TABLE S1. IUCN *Red List* data summarized for angiosperm families

Taxon	Number of records	Proportion threatened
Acanthaceae	88	0.77
Achariaceae	17	0.76
Actinidiaceae	42	0.64
Adoxaceae	12	0.75
Aizoaceae	37	0.35
Akaniaceae	1	1.00
Alismataceae	1	1.00
Alliaceae	21	0.76
Alstroemeriaceae	14	0.86
Altingiaceae	1	0.00
Amaranthaceae	31	0.81
Anacardiaceae	105	0.75
Ancistrocladaceae	1	1.00
Anisophylleaceae	17	0.76
Annonaceae	227	0.73
Apiaceae	24	0.75
Apocynaceae	176	0.75
Aquifoliaceae	97	0.69
Araceae	89	0.73
Araliaceae	165	0.82
Arecaceae	323	0.74
Argophyllaceae	1	0.00
Aristolochiaceae	17	1.00

Asparagaceae	29	0.52
Asteliaceae	1	1.00
Asteraceae	452	0.75
Asteropeiaceae	8	0.75
Atherospermataceae	1	0.00
Balanopaceae	1	0.00
Balsaminaceae	10	0.90
Begoniaceae	50	0.90
Berberidaceae	32	0.88
Betulaceae	20	0.65
Bignoniaceae	38	0.87
Bixaceae	1	1.00
Bonnetiaceae	13	0.92
Boraginaceae	66	0.73
Brassicaceae	42	0.88
Bromeliaceae	149	0.79
Brunelliaceae	19	0.89
Burmanniaceae	4	1.00
Burseraceae	88	0.60
Buxaceae	7	0.71
Byblidaceae	5	0.20
Cactaceae	147	0.77
Calceolariaceae	28	0.82
Calyceraceae	1	1.00
Campanulaceae	150	0.91
Canellaceae	6	1.00
Cannabaceae	7	0.57

Caprifoliaceae	16	0.69
Cardiopteridaceae	1	0.00
Caricaceae	7	0.71
Caryocaraceae	5	1.00
Caryophyllaceae	32	0.81
Casuarinaceae	2	0.50
Celastraceae	86	0.72
Cephalotaceae	1	1.00
Cercidiphyllaceae	1	0.00
Chloranthaceae	4	1.00
Chrysobalanaceae	56	0.89
Cistaceae	1	1.00
Clethraceae	7	0.43
Clusiaceae	132	0.69
Colchicaceae	1	0.00
Combretaceae	36	0.83
Commelinaceae	3	1.00
Connaraceae	9	0.78
Convolvulaceae	12	0.75
Cornaceae	17	0.76
Costaceae	2	1.00
Crassulaceae	8	0.13
Crypteroniaceae	1	1.00
Cucurbitaceae	5	0.80
Cunoniaceae	34	0.65
Cyclanthaceae	20	0.80
Cyperaceae	18	0.83

Degeneriaceae	2	0.50
Didiereaceae	1	0.00
Dilleniaceae	8	1.00
Dioscoreaceae	5	0.60
Dipterocarpaceae	384	0.97
Dirachmaceae	2	1.00
Droseraceae	1	1.00
Ebenaceae	101	0.75
Elaeagnaceae	2	1.00
Elaeocarpaceae	42	0.71
Ericaceae	28	0.68
Eriocaulaceae	5	0.80
Eucommiaceae	1	0.00
eudicots	1	0.00
Euphorbiaceae	332	0.80
Eupteleaceae	1	0.00
Fabaceae	738	0.81
Fagaceae	80	0.71
Frankeniaceae	1	1.00
Gentianaceae	36	0.69
Geraniaceae	9	1.00
Gesneriaceae	92	0.85
Gomortegaceae	1	1.00
Goodeniaceae	5	0.80
Grossulariaceae	5	0.80
Gunneraceae	1	1.00
Hamamelidaceae	11	0.73

Heliconiaceae	16	0.94
Hernandiaceae	12	0.58
Hoplestigmataceae	1	1.00
Huaceae	1	1.00
Humiriaceae	5	1.00
Hypericaceae	14	0.64
Icacinaceae	6	1.00
Iridaceae	6	0.17
Irvingiaceae	2	0.00
Ixonanthaceae	2	1.00
Juglandaceae	20	0.70
Juncaceae	1	0.00
Kirkiaceae	1	0.00
Lacistemataceae	1	0.00
Lamiaceae	99	0.76
Lamiales	1	0.00
Lauraceae	266	0.75
Lecythidaceae	100	0.82
Liliaceae	5	1.00
Linaceae	3	1.00
Loasaceae	16	0.88
Loganiaceae	24	0.58
Loranthaceae	20	0.90
Lythraceae	12	0.75
Maesaceae	1	1.00
Magnoliaceae	60	0.93
Malpighiaceae	17	0.94

Malvaceae	201	0.86
Marantaceae	26	0.77
Marcgraviaceae	3	1.00
Melastomataceae	318	0.85
Meliaceae	210	0.70
Melianthaceae	2	1.00
Menispermaceae	10	0.80
Metteniusaceae	4	1.00
Molluginaceae	1	0.00
Monimiaceae	15	0.80
Moraceae	86	0.63
Moringaceae	1	1.00
Myodocarpaceae	2	1.00
Myricaceae	4	0.75
Myristicaceae	218	0.71
Myrsinaceae	103	0.77
Myrtaceae	328	0.80
Nepenthaceae	77	0.66
Nothofagaceae	9	0.67
Nyctaginaceae	14	0.64
Ochnaceae	24	0.75
Olacaceae	13	0.69
Oleaceae	25	0.80
Onagraceae	12	0.67
Opiliaceae	2	1.00
Orchidaceae	150	0.97
Orobanchaceae	7	0.57

Oxalidaceae	11	0.55
Pandanaceae	25	0.88
Passifloraceae	29	0.76
Paulowniaceae	1	1.00
Pedaliaceae	1	0.00
Pennantiaceae	1	1.00
Pentaphylacaeae	67	0.82
Peridiscaceae	1	1.00
Phyllanthaceae	91	0.76
Picramniaceae	2	1.00
Picrodendraceae	4	0.75
Piperaceae	110	0.92
Pittosporaceae	32	0.78
Plantaginaceae	6	0.50
Plumbaginaceae	6	0.50
Poaceae	66	0.65
Podostemaceae	6	1.00
Polemoniaceae	2	1.00
Polygalaceae	17	0.82
Polygonaceae	21	0.86
Portulacaceae	4	0.75
Proteaceae	49	0.80
Putranjavaceae	26	0.92
Ranunculaceae	6	0.83
Resedaceae	2	0.00
Rhamnaceae	31	0.77
Rhizophoraceae	26	0.85

Rhynchocalycaceae	1	1.00
Rosaceae	106	0.75
Rubiaceae	430	0.87
Rutaceae	129	0.87
Sabiaceae	4	1.00
Salicaceae	95	0.83
Salvadoraceae	1	0.00
Santalaceae	8	0.75
Sapindaceae	124	0.85
Sapotaceae	325	0.75
Sarcolaenaceae	16	0.63
Sarraceniaceae	7	0.29
Schisandraceae	4	0.50
Schlegeliaceae	1	1.00
Scrophulariaceae	17	0.59
Simaroubaceae	10	0.80
Siparunaceae	8	0.88
Solanaceae	73	0.67
Staphyleaceae	1	1.00
Stemonuraceae	3	1.00
Stilbaceae	1	0.00
Styracaceae	19	0.89
Surianaceae	1	1.00
Symplocaceae	39	0.92
Tamaricaceae	1	0.00
Tapisciaceae	3	0.67
Tetramelaceae	2	0.00

Theaceae	24	0.96
Theophrastaceae	6	0.83
Thymelaeaceae	37	0.84
Ticodendraceae	1	1.00
Torricelliaceae	7	0.57
Triuridaceae	1	1.00
Tropaeolaceae	8	1.00
Ulmaceae	9	0.78
Urticaceae	22	0.68
Verbenaceae	21	0.81
Violaceae	32	0.78
Vitaceae	7	0.00
Vochysiaceae	4	1.00
Winteraceae	5	1.00
Xanthorrhoeaceae	25	0.48
Xyridaceae	1	1.00
Zingiberaceae	4	0.25
Zygophyllaceae	9	0.56
