# Supplementary material for: Extinction Risk and Diversification Are Linked in a Plant Biodiversity Hotspot
Source: PLoS Biol. 2011 May 24;9(5):e1000620. doi: 10.1371/journal.pbio.1000620 (PMC3101198; doi:10.1371/journal.pbio.1000620)
Supplement: Table S2 — IUCN Red List data summarized for angiosperm orders. (0.02 MB PDF) [file pbio.1000620.s003.pdf]

TABLE S2. IUCN *Red List* data summarized for angiosperm orders

Taxon	Number of records	Proportion threatened
Alismatales	90	0.73
Apiales	231	0.80
Aquifoliales	101	0.69
Arecales	323	0.74
Asparagales	232	0.82
Asterales	609	0.79
Austrobaileyales	4	0.50
Brassicales	54	0.81
Canellales	11	1.00
Caryophyllales	383	0.70
Celastrales	86	0.72
Commelinales	3	1.00
Cornales	33	0.82
Crossosomatales	1	1.00
Cucurbitales	74	0.84
Dioscoreales	9	0.78
Dipsacales	28	0.71
Ericales	884	0.77
Fabales	756	0.81
Fagales	136	0.70
Garryales	1	0.00
Gentianales	666	0.82
Geraniales	11	1.00

Gunnerales	1	1.00
Lamiales	431	0.77
Laurales	303	0.75
Liliales	20	0.85
Magnoliales	507	0.74
Malpighiales	934	0.79
Malvales	648	0.92
Myrtales	712	0.82
Oxalidales	97	0.68
Pandanales	46	0.85
Piperales	127	0.93
Poales	240	0.75
Proteales	49	0.80
Ranunculales	49	0.84
Rosales	265	0.71
Santalales	43	0.81
Sapindales	667	0.76
Saxifragales	26	0.50
Solanales	85	0.68
Zingiberales	48	0.79
