# Supplementary material for: Extinction Risk and Diversification Are Linked in a Plant Biodiversity Hotspot
Source: PLoS Biol. 2011 May 24;9(5):e1000620. doi: 10.1371/journal.pbio.1000620 (PMC3101198; doi:10.1371/journal.pbio.1000620)
Supplement: Table S3 — South African families. (0.03 MB PDF) [file pbio.1000620.s004.pdf]

TABLE S3. South African families

Taxon	number of records	proportion threatened	p-value
Acanthaceae	266	0.05	0.00
Achariaceae	6	0.00	0.39
Actiniopteridaceae	2	0.00	0.69
Adiantaceae	46	0.02	0.01
Aizoaceae	1420	0.18	0.01
Alismataceae	2	0.00	0.71
Alliaceae	254	0.30	0.00
Amaranthaceae	132	0.01	0.00
Anacardiaceae	104	0.10	0.09
Annonaceae	11	0.00	0.14
Aphloiaceae	1	0.00	0.87
Apiaceae	201	0.12	0.23
Apocynaceae	647	0.17	0.34
Aponogetonaceae	9	0.33	0.22
Aquifoliaceae	1	0.00	0.83
Araceae	21	0.14	0.92
Araliaceae	16	0.00	0.06
Arecaceae	6	0.33	0.28
Asparagaceae	657	0.17	0.35
Aspleniaceae	39	0.00	0.00
Asteraceae	2082	0.10	0.00
Azollaceae	1	0.00	0.85
Balanophoraceae	2	0.00	0.70
Balsaminaceae	3	0.33	0.45

Begoniaceae	5	0.40	0.21
Bignoniaceae	10	0.10	0.68
Blechnaceae	10	0.00	0.19
Boraginaceae	83	0.16	1.00
Brassicaceae	166	0.06	0.00
Bruniaceae	70	0.29	0.00
Burmanniaceae	1	0.00	0.84
Burseraceae	18	0.00	0.04
Buxaceae	2	0.00	0.73
Cactaceae	1	0.00	0.85
Campanulaceae	338	0.10	0.00
Canellaceae	1	1.00	0.15
Cannabaceae	5	0.00	0.41
Caprifoliaceae	24	0.13	0.70
Caryophyllaceae	44	0.02	0.00
Celastraceae	86	0.12	0.31
Ceratophyllaceae	3	0.00	0.59
Chrysobalanaceae	5	0.00	0.42
Clusiaceae	2	0.00	0.71
Colchicaceae	85	0.11	0.20
Combretaceae	32	0.03	0.03
Commelinaceae	34	0.06	0.10
Connaraceae	1	0.00	0.83
Convolvulaceae	85	0.00	0.00
Crassulaceae	330	0.08	0.00
Cucurbitaceae	65	0.05	0.01
Cunoniaceae	2	0.00	0.69

Cupressaceae	3	0.67	0.07
Curtisiaceae	1	1.00	0.15
Cyatheaceae	2	0.00	0.69
Cymodoceaceae	2	0.00	0.70
Cyperaceae	427	0.05	0.00
Cytinaceae	3	0.33	0.46
Davalliaceae	2	0.00	0.73
Dennstaedtiaceae	8	0.00	0.26
Dioscoreaceae	13	0.31	0.15
Droseraceae	20	0.00	0.04
Dryopteridaceae	21	0.00	0.03
Ebenaceae	52	0.00	0.00
Elatinaceae	9	0.00	0.20
Equisetaceae	1	0.00	0.86
Ericaceae	804	0.24	0.00
Eriocaulaceae	11	0.00	0.16
eudicots	13	0.00	0.10
Euphorbiaceae	367	0.07	0.00
Fabaceae	1578	0.19	0.00
Flagellariaceae	1	0.00	0.82
Frankeniaceae	2	0.00	0.71
Geissolomataceae	1	0.00	0.83
Gentianaceae	79	0.04	0.00
Geraniaceae	289	0.10	0.00
Gesneriaceae	63	0.25	0.03
Gisekiaceae	3	0.00	0.58
Gleicheniaceae	3	0.00	0.58

Goodeniaceae	2	0.00	0.71
Grammitidaceae	1	0.00	0.84
Grubbiaceae	6	0.00	0.36
Gunneraceae	1	0.00	0.85
Haemodoraceae	9	0.11	0.84
Haloragaceae	1	0.00	0.85
Hamamelidaceae	3	0.00	0.61
Hernandiaceae	1	0.00	0.83
Hydnoraceae	3	0.00	0.60
Hydrocharitaceae	10	0.00	0.19
Hydrostachyaceae	1	1.00	0.15
Hymenophyllaceae	11	0.00	0.17
Hypericaceae	7	0.00	0.32
Hypoxidaceae	79	0.13	0.44
Icacinaceae	8	0.13	0.89
Iridaceae	1132	0.30	0.00
Isoetaceae	9	0.78	0.00
Iteaceae	1	0.00	0.85
Juncaceae	16	0.00	0.05
Juncaginaceae	2	0.00	0.70
Kirkiaceae	2	0.00	0.70
Lamiaceae	257	0.06	0.00
Lamiales	10	0.00	0.17
Lanariaceae	1	0.00	0.85
Lauraceae	12	0.50	0.01
Lentibulariaceae	18	0.00	0.05
Linaceae	15	0.00	0.07

Loasaceae	1	0.00	0.85
Loganiaceae	10	0.00	0.17
Lomariopsidaceae	9	0.00	0.21
Loranthaceae	48	0.00	0.00
Lycopodiaceae	10	0.00	0.19
Lythraceae	26	0.04	0.09
Maesaceae	2	0.00	0.72
Malpighiaceae	7	0.00	0.31
Malvaceae	323	0.04	0.00
Marattiaceae	1	0.00	0.85
Marsileaceae	14	0.14	0.94
Melastomataceae	9	0.00	0.23
Meliaceae	13	0.15	0.95
Melianthaceae	14	0.00	0.08
Menispermaceae	13	0.00	0.11
Menyanthaceae	6	0.17	0.89
Molluginaceae	97	0.05	0.00
Monimiaceae	1	0.00	0.84
Montiniaceae	1	0.00	0.81
Moraceae	30	0.00	0.01
Musaceae	1	0.00	0.85
Myricaceae	10	0.10	0.72
Myrsinaceae	9	0.11	0.82
Myrtaceae	24	0.21	0.52
Neuradaceae	7	0.00	0.30
Nyctaginaceae	11	0.00	0.15
Nymphaeaceae	4	0.00	0.51

Ochnaceae	13	0.08	0.49
Olacaceae	4	0.00	0.51
Oleaceae	26	0.00	0.01
Oleandraceae	3	0.00	0.59
Oliniaceae	6	0.00	0.36
Onagraceae	8	0.00	0.25
Ophioglossaceae	14	0.07	0.43
Orchidaceae	474	0.18	0.10
Orobanchaceae	61	0.07	0.02
Osmundaceae	2	0.00	0.74
Oxalidaceae	185	0.19	0.20
Papaveraceae	7	0.00	0.31
Parkeriaceae	1	0.00	0.82
Passifloraceae	25	0.16	0.92
Pedaliaceae	18	0.00	0.06
Penaeaceae	27	0.30	0.07
Phrymaceae	1	0.00	0.84
Phyllanthaceae	37	0.03	0.02
Phytolaccaceae	6	0.00	0.36
Picrodendraceae	2	0.00	0.69
Piperaceae	6	0.00	0.37
Pittosporaceae	1	0.00	0.85
Plantaginaceae	20	0.00	0.03
Plumbaginaceae	20	0.25	0.29
Poaceae	739	0.04	0.00
Podocarpaceae	4	0.00	0.49
Podostemaceae	2	0.00	0.70

Polygalaceae	188	0.19	0.32
Polygonaceae	33	0.03	0.04
Polypodiaceae	14	0.00	0.10
Pontederiaceae	2	0.00	0.70
Portulacaceae	52	0.08	0.09
Potamogetonaceae	10	0.10	0.68
Proteaceae	377	0.68	0.00
Psilotaceae	1	0.00	0.83
Pteridaceae	8	0.00	0.25
Putranjavaceae	6	0.00	0.36
Ranunculaceae	26	0.12	0.57
Resedaceae	3	0.00	0.59
Restionaceae	333	0.18	0.27
Rhamnaceae	178	0.21	0.08
Rhizophoraceae	13	0.15	0.94
Rhynchocalycaceae	1	1.00	0.17
Roridulaceae	2	0.00	0.72
Rosaceae	160	0.20	0.12
Rubiaceae	241	0.04	0.00
Ruppiaceae	2	0.00	0.71
Rutaceae	300	0.36	0.00
Salicaceae	23	0.04	0.14
Salvadoraceae	4	0.00	0.49
Santalaceae	133	0.05	0.00
Sapindaceae	25	0.04	0.11
Sapotaceae	15	0.07	0.35
Schizaeaceae	11	0.00	0.17

Scrophulariaceae	713	0.10	0.00
Selaginellaceae	6	0.00	0.35
Smilacaceae	1	0.00	0.83
Solanaceae	52	0.02	0.00
Stangeriaceae	1	1.00	0.15
Stilbaceae	26	0.15	0.99
Strelitziaceae	6	0.17	0.85
Tamaricaceae	1	0.00	0.83
Tecophilaeaceae	9	0.11	0.80
Thelypteridaceae	14	0.07	0.39
Theophrastaceae	2	0.00	0.71
Thurniaceae	1	0.00	0.81
Thymelaeaceae	180	0.17	0.63
Typhaceae	1	0.00	0.84
Urticaceae	20	0.00	0.03
Vahliaceae	5	0.00	0.43
Velloziaceae	9	0.00	0.24
Verbenaceae	30	0.00	0.00
Violaceae	8	0.00	0.24
Vitaceae	47	0.04	0.02
Vittariaceae	1	0.00	0.85
Woodsiaceae	7	0.14	0.98
Xanthorrhoeaceae	512	0.26	0.00
Xyridaceae	8	0.00	0.27
Zamiaceae	37	0.89	0.00
Zingiberaceae	1	1.00	0.16
Zosteraceae	1	0.00	0.86

Zygophyllaceae	50	0.04	0.01
