# Supplementary material for: Extinction Risk and Diversification Are Linked in a Plant Biodiversity Hotspot
Source: PLoS Biol. 2011 May 24;9(5):e1000620. doi: 10.1371/journal.pbio.1000620 (PMC3101198; doi:10.1371/journal.pbio.1000620)
Supplement: Table S4 — South African orders. (0.02 MB PDF) [file pbio.1000620.s005.pdf]

TABLE S4. South African orders

Taxon	number of records	proportion threatened	p-value
Alismatales	59	0.12	0.43
Apiales	218	0.11	0.07
Aquifoliales	1	0.00	0.85
Arecales	6	0.33	0.31
Asparagales	3118	0.24	0.00
Asterales	2428	0.10	0.00
Brassicales	173	0.06	0.00
Canellales	1	1.00	0.16
Caryophyllales	1842	0.15	0.49
Celastrales	86	0.12	0.29
Ceratophyllales	3	0.00	0.61
Commelinales	45	0.07	0.09
Cornales	9	0.22	0.62
Cucurbitales	70	0.07	0.03
Dioscoreales	14	0.29	0.20
Dipsacales	24	0.13	0.73
Ericales	892	0.22	0.00
Fabales	1766	0.19	0.00
Fagales	10	0.10	0.70
Gentianales	977	0.12	0.00
Geraniales	303	0.09	0.00
Gunnerales	1	0.00	0.84
Lamiales	1519	0.08	0.00
Laurales	14	0.43	0.02

Liliales	86	0.10	0.19
Magnoliales	11	0.00	0.14
Malpighiales	547	0.06	0.00
Malvales	510	0.08	0.00
Myrtales	133	0.12	0.22
Oxalidales	188	0.19	0.26
Pandanales	9	0.00	0.22
Piperales	9	0.00	0.22
Poales	1537	0.07	0.00
Proteales	377	0.68	0.00
Ranunculales	46	0.07	0.08
Rosales	393	0.18	0.32
Santalales	185	0.04	0.00
Sapindales	462	0.26	0.00
Saxifragales	335	0.08	0.00
Solanales	138	0.01	0.00
Zingiberales	8	0.25	0.48
