# Supplementary material for: Extinction Risk and Diversification Are Linked in a Plant Biodiversity Hotspot
Source: PLoS Biol. 2011 May 24;9(5):e1000620. doi: 10.1371/journal.pbio.1000620 (PMC3101198; doi:10.1371/journal.pbio.1000620)
Supplement: Table S5 — South African APG taxonomic class 4. (0.02 MB PDF) [file pbio.1000620.s006.pdf]

TABLE S5. South African APG taxonomic class 4

Taxon	number of records	proportion threatened	p-value
asterids	901	0.22	0.00
Caryophyllales	1842	0.15	0.50
commelinids	1596	0.08	0.00
euasterids_I	2730	0.10	0.00
euasterids_II	2741	0.11	0.00
eurosids_I	3110	0.16	0.92
eurosids_II	1145	0.15	0.69
magnoliids	35	0.20	0.51
noncommelinids	3286	0.24	0.00
rosids	485	0.09	0.00
Santalales	185	0.04	0.00
Saxifragales	335	0.08	0.00
