# Supplementary material for: Extinction Risk and Diversification Are Linked in a Plant Biodiversity Hotspot
Source: PLoS Biol. 2011 May 24;9(5):e1000620. doi: 10.1371/journal.pbio.1000620 (PMC3101198; doi:10.1371/journal.pbio.1000620)
Supplement: Table S6 — South African APG taxonomic class 5. (0.02 MB PDF) [file pbio.1000620.s007.pdf]

TABLE S6. South African APG taxonomic class 5

Taxon	number of records	proportion threatened	p-value
asterids	6372	0.12	0.00
coreeudicots	2363	0.13	0.00
eudicots	438	0.59	0.00
magnoliids	35	0.20	0.47
monocots	4882	0.18	0.00
rosids	4740	0.15	0.12
