# Supplementary material for: Extinction Risk and Diversification Are Linked in a Plant Biodiversity Hotspot
Source: PLoS Biol. 2011 May 24;9(5):e1000620. doi: 10.1371/journal.pbio.1000620 (PMC3101198; doi:10.1371/journal.pbio.1000620)
Supplement: Table S7 — UK families. (0.03 MB PDF) [file pbio.1000620.s008.pdf]

**TABLE S7. UK families**

| Taxon | number of records | proportion threatened | p-value |
|---|---|---|---|
| Zosteraceae | 2 | 0.500 | 0.428 |
| Violaceae | 20 | 0.200 | 0.500 |
| Verbenaceae | 1 | 0.000 | 0.791 |
| Urticaceae | 4 | 0.000 | 0.330 |
| Ulmaceae | 3 | 0.000 | 0.424 |
| Typhaceae | 6 | 0.000 | 0.179 |
| Tofieldiaceae | 1 | 0.000 | 0.760 |
| Thymelaeaceae | 2 | 0.500 | 0.396 |
| Theophrastaceae | 1 | 0.000 | 0.763 |
| Solanaceae | 4 | 0.250 | 0.649 |
| Scrophulariaceae | 8 | 0.000 | 0.110 |
| Scheuchzeriaceae | 1 | 0.000 | 0.768 |
| Saxifragaceae | 14 | 0.357 | 0.214 |
| Sapindaceae | 1 | 0.000 | 0.757 |
| Santalaceae | 1 | 0.000 | 0.754 |
| Salicaceae | 24 | 0.125 | 0.146 |
| Ruppiaceae | 2 | 0.000 | 0.609 |
| Rubiaceae | 20 | 0.150 | 0.287 |
| Rosaceae | 104 | 0.327 | 0.016 |
| Rhamnaceae | 2 | 0.000 | 0.590 |
| Resedaceae | 2 | 0.000 | 0.570 |
| Ranunculaceae | 42 | 0.167 | 0.209 |
| Primulaceae | 6 | 0.167 | 0.563 |
| Potamogetonaceae | 33 | 0.455 | 0.010 |

| | | | |
|---|---|---|---|
| Portulacaceae | 3 | 0.000 | 0.467 |
| Polygonaceae | 39 | 0.154 | 0.152 |
| Polygalaceae | 4 | 0.000 | 0.343 |
| Polemoniaceae | 1 | 0.000 | 0.775 |
| Poaceae | 138 | 0.094 | 0.000 |
| Plumbaginaceae | 8 | 0.250 | 0.615 |
| Plantaginaceae | 46 | 0.087 | 0.005 |
| Parnassiaceae | 1 | 0.000 | 0.771 |
| Papaveraceae | 26 | 0.154 | 0.278 |
| Oxalidaceae | 1 | 0.000 | 0.786 |
| Orobanchaceae | 37 | 0.351 | 0.066 |
| Orchidaceae | 52 | 0.404 | 0.004 |
| Onagraceae | 14 | 0.000 | 0.019 |
| Oleaceae | 2 | 0.000 | 0.588 |
| Nymphaeaceae | 3 | 0.000 | 0.456 |
| Nartheciaceae | 1 | 0.000 | 0.776 |
| Myrsinaceae | 11 | 0.000 | 0.048 |
| Myricaceae | 1 | 0.000 | 0.759 |
| Molluginaceae | 1 | 1.000 | 0.257 |
| Menyanthaceae | 2 | 0.000 | 0.608 |
| Melanthiaceae | 1 | 0.000 | 0.741 |
| Malvaceae | 7 | 0.000 | 0.153 |
| Lythraceae | 3 | 0.333 | 0.518 |
| Loranthaceae | 1 | 0.000 | 0.769 |
| Linaceae | 5 | 0.000 | 0.260 |
| Liliaceae | 4 | 0.750 | 0.030 |
| Lentibulariaceae | 8 | 0.125 | 0.406 |

| | | | |
|---|---|---|---|
| Lamiaceae | 48 | 0.313 | 0.135 |
| Juncaginaceae | 2 | 0.000 | 0.584 |
| Juncaceae | 35 | 0.143 | 0.129 |
| Iridaceae | 4 | 0.250 | 0.627 |
| Hypericaceae | 12 | 0.000 | 0.046 |
| Hydrocharitaceae | 5 | 0.600 | 0.064 |
| Haloragaceae | 3 | 0.333 | 0.514 |
| Grossulariaceae | 3 | 0.000 | 0.456 |
| Geraniaceae | 15 | 0.000 | 0.015 |
| Gentianaceae | 17 | 0.412 | 0.071 |
| Frankeniaceae | 1 | 0.000 | 0.757 |
| Fagaceae | 4 | 0.000 | 0.358 |
| Fabaceae | 77 | 0.117 | 0.010 |
| Euphorbiaceae | 13 | 0.154 | 0.345 |
| Eriocaulaceae | 1 | 0.000 | 0.787 |
| Ericaceae | 27 | 0.222 | 0.553 |
| Elatinaceae | 2 | 0.000 | 0.597 |
| Elaeagnaceae | 1 | 0.000 | 0.786 |
| Droseraceae | 4 | 0.250 | 0.645 |
| Dioscoreaceae | 1 | 0.000 | 0.777 |
| Diapensiaceae | 1 | 1.000 | 0.232 |
| Cyperaceae | 131 | 0.290 | 0.071 |
| Cucurbitaceae | 1 | 0.000 | 0.755 |
| Crassulaceae | 10 | 0.100 | 0.298 |
| Cornaceae | 2 | 0.000 | 0.597 |
| Convolvulaceae | 7 | 0.143 | 0.454 |
| Colchicaceae | 1 | 0.000 | 0.770 |

| | | | |
|---|---|---|---|
| Cistaceae | 6 | 0.333 | 0.411 |
| Ceratophyllaceae | 2 | 0.000 | 0.609 |
| Celastraceae | 1 | 0.000 | 0.767 |
| Caryophyllaceae | 76 | 0.355 | 0.006 |
| Caprifoliaceae | 14 | 0.143 | 0.344 |
| Cannabaceae | 1 | 0.000 | 0.769 |
| Campanulaceae | 13 | 0.308 | 0.326 |
| Butomaceae | 1 | 0.000 | 0.765 |
| Brassicaceae | 69 | 0.130 | 0.030 |
| Boraginaceae | 21 | 0.143 | 0.227 |
| Betulaceae | 6 | 0.000 | 0.188 |
| Berberidaceae | 1 | 0.000 | 0.770 |
| Balsaminaceae | 1 | 0.000 | 0.762 |
| Asteraceae | 535 | 0.303 | 0.000 |
| Asparagaceae | 13 | 0.308 | 0.321 |
| Araliaceae | 4 | 0.000 | 0.360 |
| Araceae | 7 | 0.143 | 0.500 |
| Aquifoliaceae | 1 | 0.000 | 0.778 |
| Apocynaceae | 1 | 0.000 | 0.770 |
| Apiaceae | 60 | 0.217 | 0.466 |
| Amaranthaceae | 30 | 0.233 | 0.587 |
| Alliaceae | 9 | 0.222 | 0.652 |
| Alismataceae | 8 | 0.250 | 0.591 |
| Adoxaceae | 5 | 0.000 | 0.280 |
