# Supplementary material for: Extinction Risk and Diversification Are Linked in a Plant Biodiversity Hotspot
Source: PLoS Biol. 2011 May 24;9(5):e1000620. doi: 10.1371/journal.pbio.1000620 (PMC3101198; doi:10.1371/journal.pbio.1000620)
Supplement: Table S8 — UK orders. (0.02 MB PDF) [file pbio.1000620.s009.pdf]

TABLE S8. UK orders

Taxon	number of records	proportion threatened	p-value
Solanales	11	0.182	0.486
Saxifragales	30	0.233	0.561
Sapindales	1	0.000	0.757
Santalales	2	0.000	0.583
Rosales	115	0.296	0.071
Ranunculales	69	0.159	0.105
Poales	311	0.180	0.011
Oxalidales	1	0.000	0.786
Myrtales	17	0.059	0.070
Malvales	15	0.200	0.533
Malpighiales	76	0.118	0.008
Liliales	6	0.500	0.136
Lamiales	150	0.220	0.381
Geraniales	15	0.000	0.015
Gentianales	38	0.263	0.390
Fagales	11	0.000	0.048
Fabales	81	0.111	0.008
euasterids I	21	0.143	0.227
Ericales	48	0.167	0.158
Discoreales	1	0.000	0.776
Dipsacales	19	0.105	0.165
Dioscoreales	1	0.000	0.777
Cucurbitales	1	0.000	0.755
Cornales	2	0.000	0.597

Ceratophyllales	2	0.000	0.609
Celastrales	2	0.000	0.583
Caryophyllales	162	0.272	0.121
Brassicales	71	0.127	0.023
Asterales	550	0.302	0.000
Asparagales	78	0.359	0.007
Aquifoliales	1	0.000	0.778
Apiales	64	0.203	0.369
angiosperms	3	0.000	0.456
Alismatales	62	0.355	0.016
