# Supplementary material for: Extinction Risk and Diversification Are Linked in a Plant Biodiversity Hotspot
Source: PLoS Biol. 2011 May 24;9(5):e1000620. doi: 10.1371/journal.pbio.1000620 (PMC3101198; doi:10.1371/journal.pbio.1000620)
Supplement: Table S9 — UK APG taxonomic class 4. (0.02 MB PDF) [file pbio.1000620.s010.pdf]

TABLE S9. UK APG taxonomic class 4

Taxon	number of records	proportion threatened	p-value
Saxifragales	30	0.233	0.561
Santalales	2	0.000	0.583
rosids	32	0.031	0.001
noncommelinids	147	0.361	0.000
eurosids II	87	0.138	0.020
eurosids I	287	0.181	0.015
eudicots	69	0.159	0.105
euasterids II	634	0.285	0.000
euasterids I	220	0.218	0.323
commelinids	311	0.180	0.011
Caryophyllales	162	0.272	0.121
asterids	50	0.160	0.128
angiosperms	6	0.000	0.213
