# Supplementary material for: Extinction Risk and Diversification Are Linked in a Plant Biodiversity Hotspot
Source: PLoS Biol. 2011 May 24;9(5):e1000620. doi: 10.1371/journal.pbio.1000620 (PMC3101198; doi:10.1371/journal.pbio.1000620)
Supplement: Table S10 — UK APG taxonomic class 5. (0.02 MB PDF) [file pbio.1000620.s011.pdf]

TABLE S10. UK APG taxonomic class 5

Taxon	number of records	proportion threatened	p-value
rosids	406	0.160	0.000
monocots	458	0.238	0.390
eudicots	69	0.159	0.105
coreeudicots	194	0.263	0.160
asterids	904	0.262	0.003
angiosperms	6	0.000	0.213
