# Supplementary material for: Extinction Risk and Diversification Are Linked in a Plant Biodiversity Hotspot
Source: PLoS Biol. 2011 May 24;9(5):e1000620. doi: 10.1371/journal.pbio.1000620 (PMC3101198; doi:10.1371/journal.pbio.1000620)
Supplement: Table S11 — Generalized linear models of extinction risk against species richness, taxon age, and diversification (genera endemic to the Cape of South Africa), weighted by number of listed species within each genus (compare with Table 1, main text). (0.02 MB PDF) [file pbio.1000620.s012.pdf]

TABLE S11. Generalized linear models of extinction risk against species richness, taxon age and diversification (genera endemic to the Cape of South Africa), weighted by number of listed species within each genus (compare with Table 1, main text).

model	AIC	explanatory variable(s)	coefficient(s)	z	p-value
1	800.73	species richness	0.60	9.20	<0.001
2	755.04	taxon age	-0.40	-10.81	<0.001
3	791.38	diversification rate	0.00	9.72	<0.001
4	727.57	species richness	0.38	5.36	<0.001
		taxon age	-0.32	-8.30	<0.001
