# Supplementary material for: Extinction Risk and Diversification Are Linked in a Plant Biodiversity Hotspot
Source: PLoS Biol. 2011 May 24;9(5):e1000620. doi: 10.1371/journal.pbio.1000620 (PMC3101198; doi:10.1371/journal.pbio.1000620)
Supplement: Table S12 — Generalized linear models of extinction risk against species richness, taxon age, and diversification (genera endemic to the Cape of South Africa), weighted by the ratio of listed species to total clade species richness within each genus (compare with Table 1, main text). (0.02 MB PDF) [file pbio.1000620.s013.pdf]

**TABLE S12. Generalized linear models of extinction risk against species richness, taxon age and diversification (genera endemic to the Cape of South Africa), weighted by the ratio of listed species to total clade species richness within each genus (compare with Table 1, main text).**

| model | AIC | explanatory variable(s) | coefficient(s) | z | p-value |
|---|---|---|---|---|---|
| 1 | 420.99 | species richness | 0.47 | 5.62 | <0.001 |
| 2 | 394.06 | taxon age | -0.36 | -7.12 | <0.001 |
| 3 | 423.33 | diversification rate | 0.00 | 5.45 | <0.001 |
| 4 | 386.36 | species richness | 0.28 | 3.08 | 0.002 |
| | | taxon age | -0.31 | -5.73 | <0.001 |
