# Supplementary material for: Extinction Risk and Diversification Are Linked in a Plant Biodiversity Hotspot
Source: PLoS Biol. 2011 May 24;9(5):e1000620. doi: 10.1371/journal.pbio.1000620 (PMC3101198; doi:10.1371/journal.pbio.1000620)
Supplement: Table S13 — Correlation coefficients between extinction risk, species richness, and taxon age, from Mantel tests controlling for phylogenetic non-independence among genera (compare with GLMs, Tables 1, S11, and S12). (0.02 MB PDF) [file pbio.1000620.s014.pdf]

TABLE S13. Correlation coefficients between extinction risk, species richness and taxon age, from Mantel tests controlling for phylogenetic non-independence among genera (compare with GLMs, Tables 1, S11 and S12).

model	explanatory variable	constant	R	p-value
1	species richness	phylogenetic distance	0.15	<0.01
2	taxon age	phylogenetic distance	0.061	0.10
3	diversification rate	phylogenetic distance	0.28	<0.01
4	species richness	phylogenetic distance<br>taxon age	0.15	<0.001
5	species richness	phylogenetic distance<br>diversification rate	0.01	0.40
6	taxon age	phylogenetic distance<br>species richness	0.05	0.09
7	taxon age	phylogenetic distance<br>diversification rate	0.01	0.33
8	diversification rate	phylogenetic distance<br>species richness	0.29	<0.01
9	diversification rate	phylogenetic distance<br>taxon age	0.32	<0.01
